# Free and Total Amino Acids in Human Milk in Relation to Maternal and Infant Characteristics and Infant Health Outcomes: The Ulm SPATZ Health Study

**DOI:** 10.3390/nu13062009

**Published:** 2021-06-10

**Authors:** Joris H. J. van Sadelhoff, Linda P. Siziba, Lisa Buchenauer, Marko Mank, Selma P. Wiertsema, Astrid Hogenkamp, Bernd Stahl, Johan Garssen, Dietrich Rothenbacher, Jon Genuneit

**Affiliations:** 1 Division of Pharmacology, Utrecht Institute for Pharmaceutical Sciences, Faculty of Science, Utrecht University, 3584 CG Utrecht, The Netherlands; A.Hogenkamp@uu.nl (A.H.); J.Garssen@uu.nl (J.G.); 2 Pediatric Epidemiology, Department of Pediatrics, Medical Faculty, Leipzig University, 04103 Leipzig, Germany; Linda.Siziba@medizin.uni-leipzig.de (L.P.S.); Lisa.buchenauer@ufz.de (L.B.); Jon.Genuneit@medizin.uni-leipzig.de (J.G.); 3 Danone Nutricia Research, 3584 CT Utrecht, The Netherlands; Marko.MANK@danone.com (M.M.); Selma.WIERTSEMA@nutricia.com (S.P.W.); Bernd.STAHL@danone.com (B.S.); 4 Department of Chemical Biology & Drug Discovery, Utrecht Institute for Pharmaceutical Sciences, Utrecht University, 3584 CG Utrecht, The Netherlands; 5 Institute of Epidemiology and Medical Biometry, Ulm University, 89079 Ulm, Germany; Dietrich.Rothenbacher@uni-ulm.de

**Keywords:** human milk, breastfeeding, lactation, free amino acids, growth, allergy

## Abstract

Free amino acids (FAAs) are important regulators of key pathways necessary for growth, development, and immunity. Data on FAAs in human milk (HM) and their roles in infant development are limited. We investigated the levels of FAAs and total amino acids (TAA, i.e., the sum of conjugated amino acids and FAAs) in HM in relation to infant and maternal characteristics and immunological conditions. FAA and TAA levels in HM sampled at 6 weeks (*n* = 671) and 6 months (*n* = 441) of lactation were determined using high-performance liquid chromatography. Child growth was ascertained at 4–5 weeks and at 6–7 months of age. Child allergy and lower respiratory tract infections were assessed in the first years of life. Associations of amino acid (AA) levels in HM with child growth and health outcomes were determined by Spearman correlation and modified Poisson regression, respectively. Free glutamine, glutamate, and serine in 6-week HM positively correlated with infant weight gain in the first 4–5 weeks of age. Maternal pre-pregnancy weight and body mass index (BMI) were negatively correlated with free glutamine and asparagine in 6-week and 6-month HM and positively correlated with the sum of TAAs in 6-month HM, but significance was lost following confounder adjustment. Free glutamine was lower in 6-month HM of mothers with an allergy (either active or non-active). No consistent associations were found between FAAs in HM and child health outcomes. However, potential negative associations were observed between specific FAAs and the risk of food allergy. These results suggest that specific FAAs play a role in infant growth. Moreover, these findings warrant further investigations into the relation of FAAs in HM with infant health outcomes and maternal allergy.

## 1. Introduction

Human milk (HM) is the preferred source of nutrition during early infancy. It supports infant growth and development and provides protective effects against several immunological conditions in early life, including various infectious [1,2,3] and allergic diseases [4,5,6,7]. HM contains various bioactive components that could contribute to these abilities, such as specific proteins and peptides [8]. Besides these chains of conjugated amino acids (AAs), HM contains free amino acids (FAAs), which account for 4–10% of the total amino acid (TAA, i.e., the sum of FAAs and conjugated AAs) content in HM [9,10]. An adequate AA intake in early life is recognized to be critical for optimal infant growth and health. However, the current recommended AA intakes are based mostly on the average TAA intake of breastfed infants, without making a distinction between conjugated AAs and FAAs. Absorption kinetics for FAAs and conjugated AAs are different and, in contrast to conjugated AAs, FAAs can be readily recognized by receptors present on a wide variety of cells. Hence, FAAs may serve different functions in the neonate than conjugated AAs. Thus, it is of great interest to separately investigate levels of FAAs and TAAs supplied through HM and their associations with infant growth and health. These data could be used for enhancing other infant feeding regimens such as standard infant milk formulas, which currently contain 10% or less of the FAAs present in HM.

Levels of FAAs in HM are dynamic, with levels of some FAAs increasing while others decrease during the first 3–6 months of lactation [9,10,11]. These dynamics are consistent in studies across various ethnic groups and geographical locations, suggesting that FAA levels in HM are regulated and that FAAs might have physiological roles in infant development. In support, specific FAAs have been shown to exert physiological effects in vitro and in young animals [12,13,14,15,16,17]. These effects range from the promotion of intestinal development and overall growth to the direct and indirect modification of immunological mechanisms relevant for allergic sensitization and infections. Based on these findings, it can be hypothesized that FAAs in HM may play a role in infant growth, as well as in shaping defensive mechanisms against early life immunological conditions.

To date, no studies are available on the relation between FAAs in HM and immunological conditions in early life. A few studies have examined associations of FAAs in HM with infant characteristics and have found some evidence for associations of specific FAAs with infant growth [18,19], as well as with infant sex [10,18]. However, these studies had fewer than 80 mother–infant pairs, tested associations only at one timepoint during lactation, did not strictly control sample collection times and methods, and/or did not consider maternal characteristics as potential confounding factors. To overcome these research limitations, we investigated associations of infant anthropometrics, infant sex, and maternal characteristics with FAA levels in HM samples, collected in a strictly controlled sampling procedure at 6 weeks and 6 months postdelivery. Importantly, similar associations were investigated for TAA levels of each AA. These outcomes were compared to the results obtained for FAAs to evaluate whether such associations were simply AA-specific rather than determined by the AA being a FAA. Moreover, this study investigated for the first time whether FAAs in HM could be associated with the occurrence of several commonly occurring immune-related conditions in early life, including atopic dermatitis, wheeze, lower respiratory tract infections, and food allergy.

## 2. Materials and Methods

### 2.1. Study Design and Population

Data were obtained from the Ulm SPATZ Health Study, an ongoing birth cohort study that, at baseline, included 1006 newborns of 970 mothers (49% of all 1999 eligible families). Details are described elsewhere [20]. Mothers were recruited from the general population shortly after delivery in the University Medical Centre Ulm, southern Germany in the period April 2012–May 2013. Exclusion criteria were outpatient delivery, maternal age <18 years, transfer of the newborn or the mother to intensive care immediately after delivery, stillbirth, and insufficient knowledge of the German language. The ethics board of Ulm University (No. 311/11, date of approval 15 February 2012) approved the Ulm SPATZ Health study, and all mothers gave written informed consent before participating. 

### 2.2. Human Milk Sample Collection 

HM samples were collected at approximately 6 weeks and 6 months postdelivery from mothers who were still actively breastfeeding at the time and willing to provide a HM sample. Mothers were instructed to manually express or pump the first ~10 mL of HM (i.e., foremilk) between 9 a.m. and 12 p.m., after breakfast and before lunch but at least one hour after the last feeding. Mothers stored the HM samples in the refrigerator until study nurses collected the samples on the same day or the day after and delivered them to the study centre. Milk samples were then aliquoted and frozen at −80 °C. 

### 2.3. Amino Acid Analyses

HM samples were stored at −80 °C until analysis of AAs in 2019. Levels of FAAs (i.e., unbound AAs) and TAAs (i.e., unbound + conjugated AAs) were analysed by liquid chromatography as described in detail elsewhere [10]. This method omitted the detection of proline and cysteine, yielding a total of 18 detectable FAAs and the non-coded AAs taurine, ornithine, and citrulline. The measurements of TAAs required acidic hydrolysis of proteins, which enabled the detection of 15 TAAs and disabled the detection of tryptophan, cysteine, and proline. The acidic hydrolysis process also transformed asparagine into aspartate (combined referred to as Asx) and glutamine into glutamate (combined referred to as Glx), disabling the detection of these TAAs individually. All measurements were done in the same lab and by the same technician in blinded fashion, thereby ruling out systematic bias in sample analysis and/or preparation.

### 2.4. Infant and Maternal Characteristics and Anthropometric Measurements

Infant and child body weight and length were obtained from examination documentation recorded during scheduled appointments at 4–5 weeks and 6–7 months of age. Infant birth weight and length were subtracted from these measurements to obtain infant weight gain and length gain, respectively, in the first 4–5 weeks and 6–7 months of age. Maternal pre-pregnancy weight was ascertained from paper-based records of the obstetrics visit at which pregnancy was established if this took place within the first 15 weeks of pregnancy or was self-reported for mothers whose first appointment took place later in pregnancy. This information was used to calculate body mass index (BMI; calculated as mass (kg)/height (m)^2^). Other demographic, lifestyle, and birth-related data including infant sex, delivery mode, birth weight and length, maternal age, education, and smoking status (within one year prior to delivery) were collected using self-administered standardized questionnaires. Maternal allergy was self-reported and classified as mothers who reported a history of hay fever, atopic dermatitis, or asthma. These mothers could either have an active or a non-active allergy. Non-allergic mothers reported the absence of an allergic disease.

### 2.5. Infant Immune Outcome

A reported doctor’s diagnosis of wheeze in the past 12 months was assessed at 1 year, 2 years, and at each yearly follow-up by self-administered questionnaires from parents. Wheezing phenotypes were classified into three categories: transient (report of wheeze at age 1 but not at age 2 and 3), intermediate (report of wheeze at age 2 but not age 1 and 3), and persistent (report of wheeze at age 1, 2, and 3). Atopic dermatitis (AD) was assessed by separate parent and paediatrician reports of doctor-diagnosed AD assessed at 1 year. AD diagnosis was classified as parent-reported (i.e., a positive report of doctor-diagnosed AD by the parents), paediatrician-reported (i.e., a positive report of doctor-diagnosed AD by the paediatrician), or parent- and paediatrician-reported (i.e., a positive report of doctor-diagnosed AD by both the paediatrician and the parents). Children with at least one positive report of AD were defined as cases in the relevant outcome category. A reported doctor’s diagnosis of lower respiratory tract infections (including pneumonia, bronchitis, pertussis, tracheobronchitis, Krupp, bronchiolitis, and flu) and food allergy was assessed at 1 year of age by self-administered questionnaires from the children’s primary care paediatricians. 

### 2.6. Statistical Analyses

Data were checked for normal distribution using Shapiro–Wilk tests and visual inspection of histogram plots. Wilcoxon rank-sum tests were used to identify differences in AA levels between 6-week and 6-month HM samples. For this analysis, a subset of 6-week samples restricted to mothers with a 6-month sample was used. Principal component analysis (PCA) was used to evaluate correlations between individual FAAs in HM. Centred-log ratio (CLR) transformation was applied to FAA data prior to PCA analysis to account for compositionality. CLR was calculated as the natural log of the quotient of concentrations of individual FAAs over the geometric mean of all FAA concentrations within a HM sample. Following PCA, a factor analysis was used to determine specific FAA patterns in HM. Visual inspection of the scree plot and the Kaiser criterion (eigenvalue ≥1) were used to determine the number of factors to be retained. Based on this, we retained factors with variable loadings ≥|0.5| [21,22]. Infant sex differences in AA levels in 6-week and 6-month HM samples were assessed by unpaired *t*-tests. The potential impact of infant sex in changes of AA levels in HM between 6 weeks and 6 months of lactation were assessed by a mixed model analysis, with subjects as random effect and infant sex and the difference in AA levels between 6-week and 6-month HM as fixed effects. 

Associations of AAs with infant and maternal anthropometrics were assessed by Spearman correlation. Associations were further investigated using partial correlations that were adjusted for all maternal factors that were associated with levels of any FAA or TAA in 6-week or 6-month HM samples. Differences in AA levels in HM between mothers with or without an allergy (either active or non-active) were assessed by Welch’s *t*-tests. Modified Poisson regression analyses [23] were used to estimate the effects of FAA levels in HM on infant health outcomes. Risk ratios (RR) were modelled with individual FAA levels as continuous independent variables. Models were adjusted for maternal pre-pregnancy BMI, maternal age, maternal education, maternal smoking status, and infant gender, as these putative confounders were associated with ≥ 5% change in the crude estimate of the effect of any individual FAA on any infant health outcome. 

Bonferroni adjustment of the level of significance was applied to account for multiple testing, using an initial limit of α = 0.05. We investigated 21 individual FAAs as well as the sum of FAAs, and therefore the Bonferroni-adjusted level of significance for FAA data was α threshold = 0.05/22 = 0.002. A total of 15 individual TAAs were investigated as well as the sum of TAAs, hence the Bonferroni-adjusted level of significance for TAA data was α threshold = 0.05/16 = 0.003. As we consider the present study to be explorative, results with a *p*-value of <0.01 but higher than the Bonferroni-adjusted level of significance were specifically indicated in tables and figures. All *p*-values are shown unadjusted for multiple comparison. Statistical analyses were done using SAS (version 9.4, The SAS Institute, Cary, NC, USA) and R (version 3.5.1, R Foundation for Statistical Computing, Vienna, Austria). Figures were generated using GraphPad Prism (version 7.03, GraphPad Software Inc., San Diego, CA, USA) and R.

## 3. Results

### 3.1. Characteristics of Participating Mothers and Their Infants

A total of 741 and 483 mothers were actively breastfeeding at 6 weeks and 6 months postdelivery, respectively. HM samples for AA analyses were available from 671 (90.6% of breastfeeding mothers) and 441 (79.8% of breastfeeding mothers) lactating women at 6 weeks and 6 months of lactation, respectively. In total, 411 lactating women provided a HM sample both at 6 weeks and at 6 months of lactation. The characteristics of lactating women and their infants included in the present study are shown in Table 1.

### 3.2. Amino Acid Levels and Composition in Human Milk at 6 Weeks and 6 Months of Lactation

The mean levels of FAAs and TAAs in 6-week and 6-month HM samples are reported in Table 2. Wilcoxon rank sum tests showed that of the 21 FAAs, only levels of histidine, glycine, arginine, and lysine were statistically similar at 6 weeks and 6 months of lactation (Table 2). Glutamine, aspartate, and citrulline were prominently higher, while valine and tyrosine were markedly lower (all *p* < 0.001) at 6 months of lactation. All of the individual TAAs were significantly lower at 6 months compared to 6 weeks of lactation (all *p* < 0.001).

**Figure 1:**
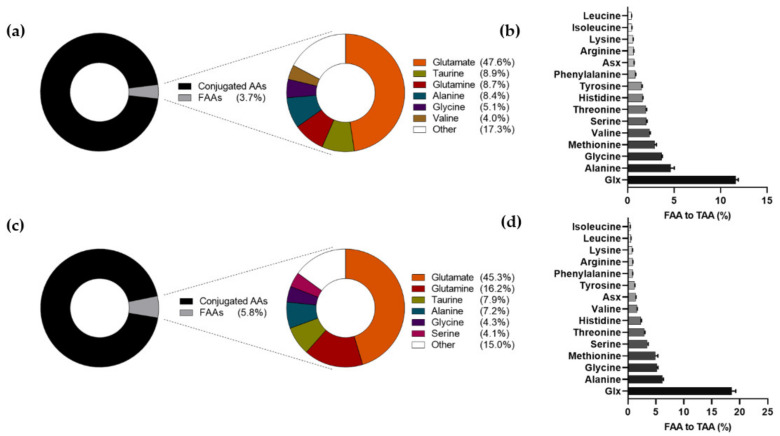
Relative abundance of individual free amino acids (FAAs) in human milk (HM) to the sum of FAAs and to the total amino acid (TAA) level of the corresponding amino acid in 6-week HM ((**a**,**b**), respectively) and 6-month HM ((**c**,**d**), respectively). Error bars show the 95% CI of the means. AA: amino acid; FAA: free amino acid; TAA: total amino acid; Asx: asparagine + aspartate; Glx: glutamate + glutamine.

The sum of FAAs accounted for 3.7% and 5.8% of the sum of TAAs in 6-week and 6-month HM, respectively (Figure 1a,c). Large differences were observed between the relative contribution of FAA to TAA of individual AAs (Figure 1b,d). The percentage of FAA to TAA was the highest for the combination of glutamine + glutamate (Glx), both in 6-week HM (11.5%) and 6-month HM (18.1%). Concentrations of free glutamine plus free glutamate accounted for approximately 56.3% and 61.5% of the sum of FAAs in 6-week and 6-month HM, respectively (Figure 1a,c).

Compositional biplots from PCA were used to evaluate potential FAA patterns based on their correlational properties. Factor analyses suggested two FAA patterns in 6-week HM, defined based on factor loadings of ≥|0.5| (Table S1, Figure S1a). The first pattern was characterised by positive scores of histidine, alanine, glutamate, glycine, threonine, serine, and citrulline and a negative score of ornithine. The second pattern comprised positive scores of lysine, leucine, and phenylalanine and a negative score of ornithine. No distinct patterns were observed at 6 months of lactation (Figure S1b).

### 3.3. Infant Sex Differences in Amino Acid Levels in Human Milk

**Figure 2:**
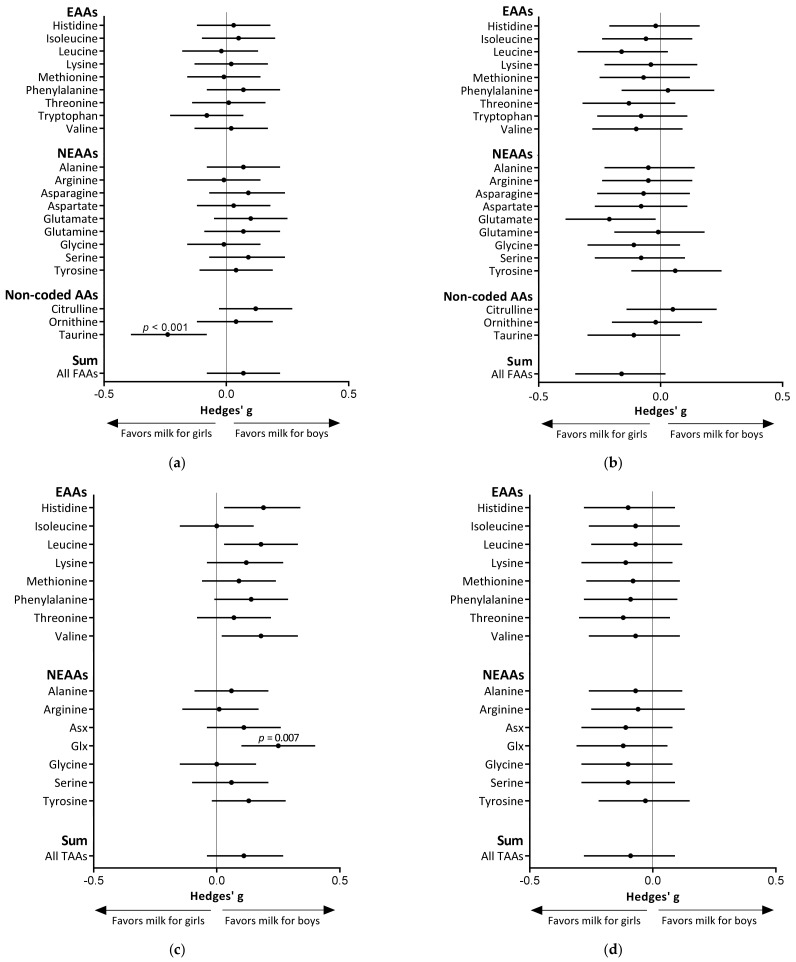
Forest plots indicating the effect sizes (Hedges’ g and 95% CI) of infant sex on free amino acid (FAA) levels in 6-week human milk (HM) (*n* = 354 for boys, *n* = 317 for girls) (**a**) and 6-month HM (*n* = 232 for boys, *n* = 209 for girls) (**b**), and on total amino acid (TAA) levels in 6-week HM (**c**) and 6-month HM (**d**). Sex differences in amino acid levels in HM were assessed by unpaired *t*-tests. The Bonferroni-adjusted level of statistical significance is α = 0.05/22 = 0.002 for FAA data and α = 0.05/16 = 0.003 for TAA data. AA: amino acid; EAA: essential amino acid; NEAA: non-essential amino acid; Asx: asparagine + aspartate; Glx: glutamate + glutamine.

HM for girls contained significantly higher levels of taurine at 6 weeks than HM for boys (*p* < 0.001; Hedges’ *g* = 0.23; Figure 2a). This difference was also observed after adjustment for maternal pre-pregnancy BMI, maternal age, and maternal allergy (*p* = 0.004), although the Bonferroni-adjusted level of significance was not reached (α threshold = 0.002). With regards to TAAs, HM for boys tended to contain higher levels of Glx (glutamine + glutamate) at 6 weeks *(p* = 0.007; Hedges’ *g* = 0.25; Figure 2c), but statistical significance was lost following Bonferroni correction (α threshold = 0.003). Absolute levels of each FAA and TAA in HM for boys and girls are shown in Table S2A,B.

At 6 weeks, all TAAs and 15 of the 21 measured FAAs were higher in HM for boys (Figure 2a,c). At 6 months, all TAAs and 18 of the 21 measured FAAs and were higher in HM for girls (Figure 2b,d). Results from post-hoc mixed model analyses showed that the increase in the sum of FAAs from 6 weeks to 6 months of lactation tended to be higher for girls (*p* = 0.091). There were no infant sex differences in the changes of the sum of TAAs over lactation (*p* = 0.185).

### 3.4. The Relation Between Maternal Characteristics and Amino Acid Levels in Human Milk

**Figure 3:**
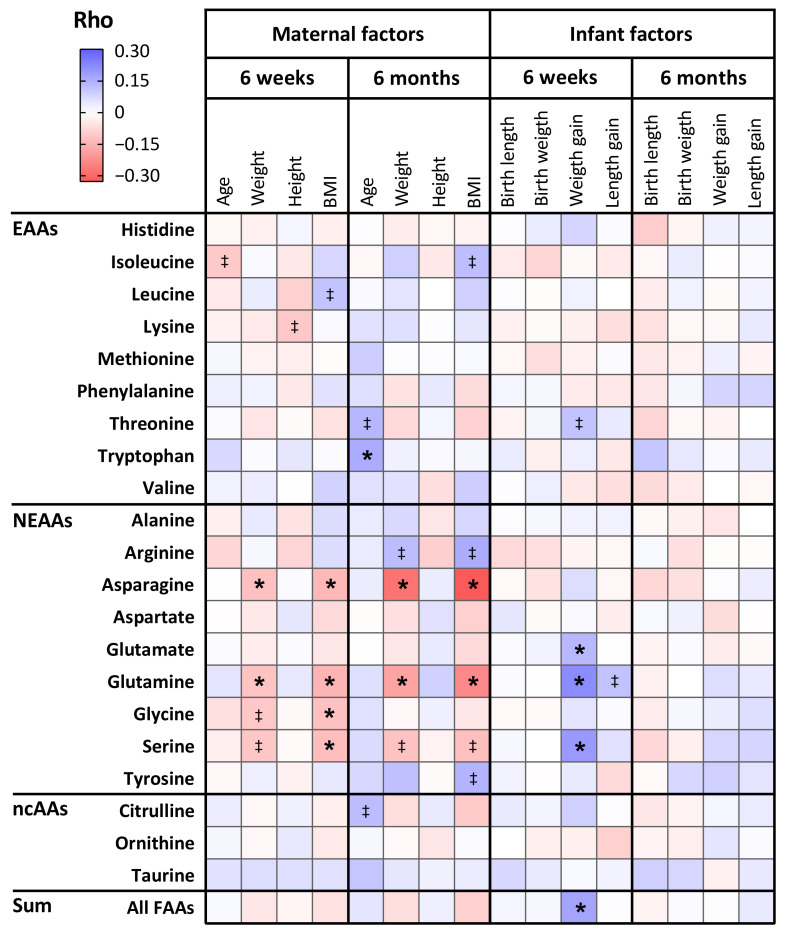
Heatmap of correlations of free amino acids (FAAs) in human milk with maternal and infant anthropometric measurements. Colouring reflects direction and magnitude of the Spearman correlation coefficients. EAA: essential amino acid; NEAA: non-essential amino acid; ncAA: non-coded amino acid; BMI: body mass index. ‡ *p* < 0.01, * *p*-value remains statistically significant following Bonferroni adjustment. Bonferroni-adjusted level of statistical significance is α = 0.05/22 = 0.002.

Negative correlations were found between maternal pre-pregnancy weight and free glutamine and asparagine in 6-week HM (*r* = −0.118 and *r* = −0.124, respectively; *p* ≤ 0.002) and in 6-month HM (*r* = −0.183 and *r* = −0.279, respectively; *p* < 0.001) (Figure 3, Table S3A). Similarly, maternal pre-pregnancy BMI negatively correlated with free glutamine and asparagine in 6-week HM (*r* = −0.151 and *r* = −0.142, respectively; *p* ≤ 0.001) and 6-month HM (*r* = −0.235 and *r* = −0.328, respectively; *p* < 0.001) (Figure 3, Table S3A). These associations, however, lost significance after adjustment for infant sex, maternal age, and maternal allergy (Table S4), except for the association of pre-pregnancy weight with free asparagine in 6-week HM (β = −0.089, *p* = 0.031). Moreover, a positive association was found between maternal age and free tryptophan in 6-month HM (*r* = 0.158, *p* = 0.001). This association was also observed after adjusting for infant sex, maternal pre-pregnancy BMI, and maternal allergy (β = 0.298, *p* = 0.034), but it did not reach the Bonferroni-adjusted level of significance.

**Figure 4:**
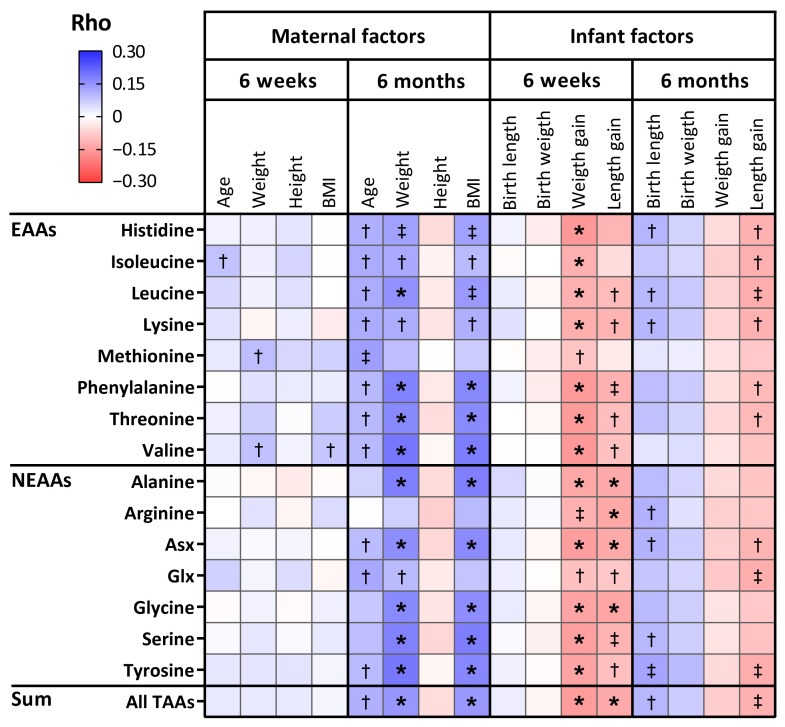
Heatmap of correlations of total amino acids (TAAs) in human milk with maternal and infant anthropometrics. Colouring reflects direction and magnitude of the Spearman correlation coefficients. EAA: essential amino acid; NEAA: non-essential amino acid; Glx: glutamate + glutamine; Asx: aspartate + asparagine; BMI: body mass index. † *p* < 0.05, ‡ *p* < 0.01, * *p*-value remains statistically significant following Bonferroni adjustment. Bonferroni-adjusted level of statistical significance is α = 0.05/16 = 0.003.

Maternal pre-pregnancy weight and BMI were positively correlated with the sum of TAAs (*r* = 0.145 and *r* = 0.143, respectively; *p* ≤ 0.003) and with most individual TAAs (Figure 4, Table S3B) in 6-month HM. However, statistical significance was lost after adjustment for infant sex, maternal age, and maternal allergy (Table S4). 

**Figure 5:**
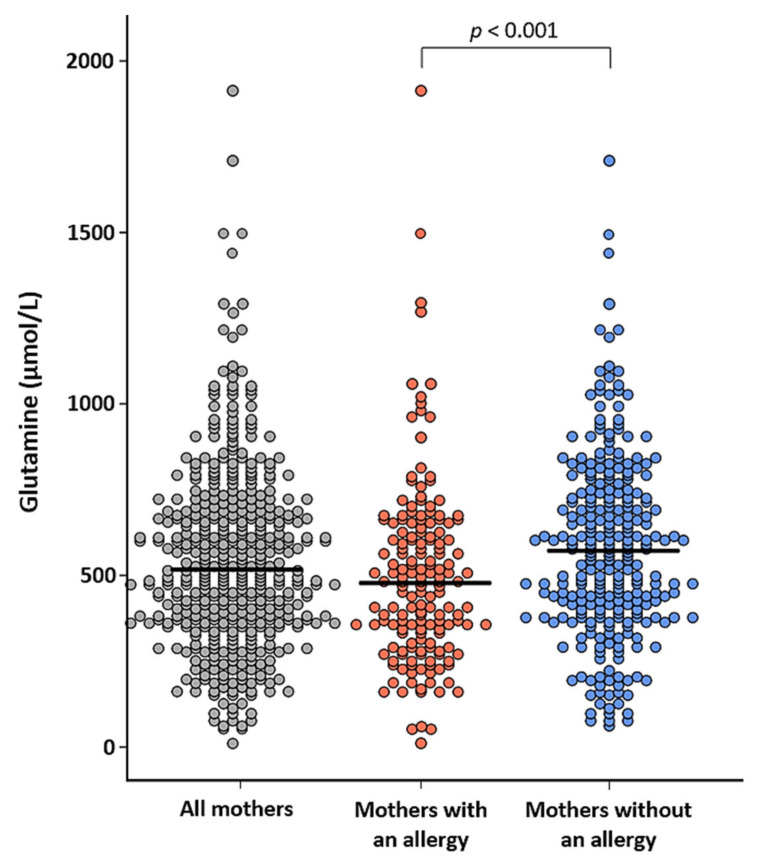
Levels of free glutamine in 6-month human milk samples from all mothers (*n* = 441) who provided a human milk sample and from mothers with (*n* = 166) or without (*n* = 274) an allergy (either active or non-active).

Levels of free glutamine were lower in 6-month HM of mothers with an allergy, compared to mothers without an allergy (mean difference = 81.1 µM, 95% CI: 30.0–132.3 µM; *p* < 0.001) (Figure 5, Table S5A,B). This difference was also observed after adjustment for pre-pregnancy BMI, maternal age, and infant sex (*p* = 0.006), although statistical significance was lost after additionally adjusting for multiple testing (α threshold = 0.002).

### 3.5. The Relation Between Amino Acid Levels in Human Milk and Infant Anthropometrics

For FAAs, positive associations were found between infant weight gain in the first 4–5 weeks of life and free glutamate, glutamine, and serine in 6-week HM (*r* = 0.123, *r* = 0.216, and *r* = 0.192, respectively; *p* < 0.001) (Figure 3, Table S3C). For serine and glutamine, these positive associations were also observed after adjustment for infant sex, maternal pre-pregnancy BMI, maternal age, and maternal allergy (β = 0.004, *p* = 0.047 and β = 0.015, *p* = 0.005, respectively) (Table S4), though these adjusted associations did not reach the Bonferroni-adjusted level of significance (α threshold = 0.002). In addition, free glutamine in 6-week HM tended to correlate with infant length gain in the first 4–5 weeks of life (*r* = 0.104, *p* = 0.003), also after adjusting for said confounders (β = 5.436, *p* = 0.088).

For TAAs, infant weight gain and length gain in the first 4–5 weeks of life was negatively associated with the sum of TAAs in 6-week HM (*r* = −0.152 and *r* = −0.132, respectively; *p* < 0.001) and with most individual TAAs (Figure 4, Table S3D). Following adjustment for infant sex, maternal pre-pregnancy BMI, maternal age, and maternal allergy, the associations of the sum of TAAs in 6-week HM with infant weight and length gain remained significant (β = −0.212 and −8.568, respectively; *p* ≤ 0.003), even after also adjusting for multiple testing (α threshold = 0.003).

### 3.6. Associations of Free Amino Acids in Human Milk and Infant Health Outcomes

A higher level of asparagine in 6-week HM was associated with a lower risk of intermediate wheeze (RR = 0.95, 95% CI: 0.92–0.98; *p* < 0.001) (Table S6A). In 6-month HM, higher levels of histidine and lysine were associated with a higher risk of intermediate wheeze (both RR = 1.04, 95% CI: 1.02–1.06; *p* < 0.002). These associations were not consistent for other wheeze outcomes (Table S6A). In addition, a higher level of free asparagine in 6-week HM was associated with a lower risk of food allergy (RR = 0.70, 95% CI: 0.58–0.84; *p* < 0.001) (Table S6C). Higher levels of arginine (RR = 0.92, 95% CI: 0.86–0.99; *p* = 0.029) and citrulline (RR = 0.85, 95% CI: 0.71–0.98; *p* = 0.047) in 6-week HM also tended to be associated with a reduced risk of food allergy, but statistical significance was lost following Bonferroni correction (α threshold = 0.002). No significant associations were found for FAAs in HM with lower respiratory tract infections and AD outcomes (Table S6B).

## 4. Discussion

The present study contributes to the understanding of AAs in HM and their potential association with infant growth and health outcomes. To our knowledge, this is the largest study to date investigating AA levels in HM in relation to infant characteristics, and the first to investigate this for FAAs and TAAs separately. Moreover, this is the first study to evaluate potential associations of FAAs with commonly occurring immunological conditions in the first years of life. 

The present study showed that the FAAs glutamine and glutamate are highly abundant in HM, both relative to other FAAs and relative to their TAA levels. This may reflect the high glutamine and glutamate needs associated with rapid cell division in neonates, particularly in the small intestines and its associated tissues [16,24]. In line with previous studies [9,10], we observed that levels of all individual TAAs were lower at 6 months compared to 6 weeks of lactation. In contrast, temporal changes of FAAs in HM between 6 weeks and 6 months of lactation were AA-specific. We also observed significant temporal changes of more individual FAA levels in comparison to the previously reported [9,10,11] limited number of FAAs. This inconsistency could be due to the larger sample size of the present study, which allows to detect smaller changes. Interestingly, we observed temporal changes in the levels of the non-coded AAs citrulline and ornithine, which, to our knowledge, have not been reported previously. These changes were most prominent for citrulline, of which levels doubled from 6 weeks to 6 months of lactation. As citrulline is recognized as a potent immunomodulator and as important for intestinal growth [25], this apparent temporal regulation of citrulline secretion in HM could be important for neonatal development.

Our results further show that some FAAs in 6-week HM could be combined into two groups based on correlational properties. Remarkably, the standard FAAs of one group were all glucogenic AAs, whereas the standard FAAs of the other group were all ketogenic AAs. Glucogenic and ketogenic AAs differ in their catabolic pathways. Glucogenic AAs can be converted into glucose, whereas ketogenic AAs can be converted into ketone bodies, both crucial metabolites for normal growth and development in the neonatal period [26,27]. The clustering of these FAAs in HM may suggest a mechanism of selective FAA secretion in HM to ensure adequate glucogenic and ketogenic AAs during this critical period of growth. However, these clusters may only be relevant in early stages of lactation, as factor analysis did not identify these same clusters in 6-month HM. 

Several factors, including infant sex, have been shown to influence AA composition in HM [10,18]. Our results suggest potential sex differences in temporal changes of the sum of FAA levels in HM over lactation. In line with our findings, previous studies indicated that levels of most FAAs were slightly higher in HM for boys in the first 3 months of lactation, but not at later stages of lactation [10,18]. These results call for replication in larger studies with a smaller time interval of longitudinally collected HM samples over lactation, to better represent the time course. In addition, the present study showed significantly higher levels of taurine in HM for girls at 6 weeks of lactation. At 6 months, taurine levels also tended to be higher in HM for girls. As infants have relatively low capacity to synthesize taurine, adequate intake of this AA in early life is considered essential for normal development [28]. Taurine intake though HM has been associated with perinatal neurodevelopment [29] and with production of taurine-conjugated bile acids in infants [28,30]. These bile acids are known to influence intestinal microbial colonization, which is reported to be different for male and female infants [31,32]. Nonetheless, whether the observed differences in taurine levels in HM for boys and girls could lead to physiological differences warrants further investigation. 

In line with previous findings [19,33,34,35,36], the present study showed that maternal pre-pregnancy BMI and weight were positively correlated with the sum of TAAs, indicative of the total protein content, and negatively correlated with free glutamine in HM. Though the direction of these associations did not change following adjustments for confounders, significance was lost. This suggests that these associations may not be solely driven by maternal pre-pregnancy BMI and weight. Interestingly, we observed significantly lower levels of free glutamine in 6-month HM samples of mothers with an allergy (either active or nonactive). During inflammatory conditions, plasma and tissue levels of free glutamine significantly drop, possibly as a result of an increased demand for glutamine by the immune system [37,38,39]. This may explain the finding of lower levels of glutamine in HM of allergic mothers. Glutamine is known to have dose-dependent immunomodulating effects in neonatal animals, including effects that could protect against allergies [16,40,41]. Granted that maternal allergic disease history is considered a risk factor for allergy development in the offspring [42], future studies should address whether a lower intake of glutamine through HM in children of allergic mothers could contribute to this association. As we could not distinguish between mothers with an active or non-active allergy, further investigation of HM of mothers with an active allergy is warranted.

Free serine, glutamine, and glutamate in 6-week HM were positively correlated with infant weight gain. Additionally, free glutamine in 6-week HM tended to be positively associated with infant length gain. Whereas associations with serine have not been previously reported, similar associations of glutamine and glutamate with infant weight and length gain have been reported in smaller studies [18,19]. Our results are in line with findings in neonatal animal studies [43,44,45], which showed increased daily body weight gain of young piglets through dietary supplementation of free glutamine, glutamate, and serine. Combined, these results suggest a causal relation of intake of these FAAs with weight gain during early life. Serine, glutamine, and glutamate have been associated with the promotion of intestinal growth and development and reduced inflammation and oxidative stress in various neonatal animal models, often in a concentration-dependent manner [12,16,44]. It is plausible that free serine, glutamine, and glutamate in HM contribute to early life growth via these mechanisms. In contrast to the positive associations of specific FAAs with infant growth, most individual TAAs and the sum of TAAs negatively correlated with infant weight and length gain in the first 4–5 weeks of life. This is somewhat inconsistent with previous studies [46,47], which have reported either positive associations of HM protein intake with infant weight and/or length gain in the first 3–6 months of life or no associations up to 12 months of age [48]. This inconsistency may be explained by differences in HM sample collection times throughout lactation.

We observed that associations of FAAs with infant and maternal factors were highly AA-specific, in contrast to associations of TAAs. Interestingly, for several AAs, including glutamine and serine, it was observed that the FAA variant positively associated with infant weight and/or length gain in the first 4–5 weeks of life, while their TAA variants revealed an inversed association. This suggests that FAAs may have different roles in infant growth and health outcomes compared to their conjugated forms. Therefore, it may be important to consider the combination of conjugated AAs and FAAs in defining an adequate AA intake in early life.

Newborns are susceptible to developing food allergies, as their underdeveloped T-helper 1-type (T_H_1) immunity results in a T-helper 2-type (T_H_2) dominant intestinal immune milieu. We observed negative associations between levels of asparagine in 6-week HM samples with the risk for food allergy. Although non-significant following correction for multiple testing, similar associations were observed with arginine and citrulline in 6-week HM samples. Interestingly, these FAAs have profound stimulating effects on intestinal barrier function and have been indicated to support T_H_1 immune responses and/or inhibit T_H_2 immune responses in neonatal animals [49,50,51]. These effects can support tolerance induction to food allergens and thus could potentially explain the observed negative associations with the risk for food allergy. We also explored correlations of FAAs with asthma, but as asthma cannot be reliably diagnosed until the age of 6 years [52] the incidence was very low (*n* = 3) in this study. Nonetheless, negative correlations were found between the risk of asthma and citrulline levels in 6-week HM samples (RR = 0.64; 95% CI: 0.53–0.79; *p* < 0.001) and 6-month HM samples (RR = 0.57, 95% CI: 0.45–0.72; *p* < 0.001). This is interesting to note because citrulline has been used effectively to prevent and control asthma in animals and humans [53,54,55]. However, granted that a small number of infants were diagnosed with food allergy (*n* = 13) and supposedly diagnosed with asthma (*n* = 3) in the present study, these results should be interpreted with caution. Therefore, future studies including larger sample sizes of children with asthma and food allergy to investigate these associations more comprehensively are warranted.

The present study has several strengths and limitations. The strengths of this study include the relatively large study population and the strictly controlled protocol of HM sample collection, which reduced the potential effect of within-feed and circadian rhythm variations on FAA levels in HM [56,57]. Another strength was that HM was the only source of AAs for most infants, as the majority of mothers exclusively fed HM at 6 weeks and 6 months postpartum (~75% and ~83%, respectively). Limitations of the current study include the lack of available information regarding the maternal diet and the lack of exclusion criteria concerning maternal chronic diseases and medication intake. These maternal factors can influence the HM composition and therefore may have confounded the results of the present study, though it remains to be elucidated whether these factors also influence the AA composition of HM. With regards to maternal diet, this influence may be limited, as FAA and TAA levels in HM are highly similar in mothers across different geographical locations with varying dietary habits [9]. Another limitation is that our analytical method did not permit quantifying the concentrations of proline and cysteine in HM samples. This is unfortunate as some studies indicate that cysteine has anti-oxidative and anti-inflammatory roles in neonates and thus may influence infant health outcomes [58,59], although these effects might be limited to critically ill neonates and pre-term infants. Further limitations include the relatively few wheeze, AD, and food allergy cases, despite the large study population. Therefore, the power of the present study to detect significant associations between levels of FAAs and clinical outcomes is limited. The application of Bonferroni correction also reduced the probability of finding a significant result based on the number of tests performed. However, this adjustment for multiple testing also reduces the risk of making extreme inferences in observational or clinical research [60]. To increase the power to detect associations with infant health outcomes, future studies could opt for high-risk cohorts consisting only of children born to allergic mothers or first-degree family members with an allergy. Furthermore, other studies could benefit from using a higher sampling frequency to better represent the time course of AAs in HM and their associations with maternal and infant characteristics. Finally, as other HM components like fatty acids and oligosaccharides may also play a role in infant development [61,62,63], future studies should focus on modelling the effects of multiple HM components simultaneously. 

## 5. Conclusions

In summary, the present study showed that changes of FAAs in HM over lactation are AA-specific, in contrast to changes of TAAs. Positive associations were observed between infant growth in the first 4–5 weeks of life and specific FAAs in HM, including glutamine and serine, while TAA levels of these AAs revealed an inversed association. No statistically significant associations were observed between AAs in HM and maternal anthropometrics following confounder adjustments. However, lower levels of free glutamine were observed in HM of mothers with an allergy (either active or non-active). This finding warrants further investigations of AA levels in HM of mothers with an active allergy. The present study observed no consistent associations between FAAs in HM and infant AD, wheeze, and lower respiratory tract infections outcomes, but suggested potential negative associations between specific FAAs and food allergy. These associations need confirmation, ideally in high-risk cohorts. Together, our findings support the hypothesis that FAAs in HM can have physiological functions in early life. Moreover, our results suggest differential physiological effects of FAAs and conjugated AAs in early life, indicating that intake of an appropriate ratio of conjugated AAs and FAAs may be relevant for optimal infant feeding.

## Figures and Tables

**Table 1:** Characteristics of the lactating women who had amino acid data available at 6 weeks and 6 months of lactation and their infants in the Ulm SPATZ Health Study.

	All 6-Week Samples (*n* = 671)		All 6-Month Samples (*n* = 441)	
	*n*	% or Mean	*n*	% or Mean
Maternal Characteristics				
Age (Years)	671	33.1	441	33.5
Pre-Pregnancy BMI				
Underweight (BMI <18.50)	15	2.3%	8	1.9%
Normal (BMI 18.50–24.99)	411	63.3%	274	64.6%
Overweight (BMI 25.00–29.99)	135	20.8%	88	20.8%
Obese (BMI ≥ 30.00)	88	13.6%	54	12.7%
Weight (kg)	617	70.5	417	69.9
Height (cm)	653	167.6	430	167.7
Parity (*n* Births of Foetus ≥ 24 weeks)				
0 births	347	51.8%	224	50.8%
≥ 1 birth	323	48.2%	217	49.2%
Maternal School Education				
Low	40	6.1%	14	3.2%
Intermediate	182	27.5%	104	24.0%
High	439	66.4%	316	72.8%
History of Smoking (Ever in Life)				
Yes	288	43.2%	169	38.5%
No	378	56.8%	270	61.5%
Maternal Allergy (Either Active or Non-Active)				
Yes	225	33.7%	166	37.7%
No	443	66.3%	274	62.3%
Exclusive Breastfeeding at the Time of Sample Collection				
Yes	502	74.8%	366	83.0%
No	169	25.2%	75	17.0%
Infant Characteristics				
Sex				
Boys	354	52.8%	232	52.6%
Girls	317	47.2%	209	47.4%
Gestational Age at Birth				
Early (37–38 weeks)	239	35.6%	148	33.6%
Full (39–40 weeks)	352	52.5%	240	54.4%
Late (41–42 weeks)	80	11.9%	53	12.0%
Method of Birth				
Vaginal spontaneous	455	67.9%	312	70.7%
Elective caesarean	67	10.0%	36	8.2%
Emergency caesarean	86	12.8%	52	11.8%
Vaginal assisted	62	9.3%	41	9.3%
Weight				
Weight at birth (g)	670	3212.5	441	3250.1
Weight at 4–5 weeks (g)	594	4365.5	416	4372.6
Weight at 6–7 months (g)	554	7852.0	385	7849.1
Length				
Length at birth (cm)	669	51.1	440	51.1
Length at 6 weeks (cm)	592	62.9	416	62.9
Length at 6 months (cm)	553	68.8	384	68.9
Atopic Dermatitis (AD)				
Parent-reported diagnosis	44	7.9%	27	7.0%
Paediatrician-reported diagnosis	64	12.6%	37	10.5%
Parent- and paediatrician-reported diagnosis	35	6.2%	20	5.1%
Wheeze Phenotypes				
Transient wheeze	47	12.1%	30	11.2%
Persistent wheeze	31	8.0%	18	6.7%
Intermediate wheeze	31	8.0%	27	10.0%
Lower respiratory tract infections	151	30.0%	108	31.0%
Food allergy	13	2.6%	7	2.0%

Note that sums may not add up to the total number of participants as percentages exclude missing data.

**Table 2:** Free amino acid and total amino acid levels in human milk sampled at 6 weeks and 6 months of lactation.

AA	FAAs				TAAs			
	6 Weeks (*n* = 671)	6 Weeks Restricted (*n* = 411)	6 Months (*n* = 441)	*p*-Value ^1^	6 Weeks (*n* = 671)	6 Weeks Restricted (*n* = 411)	6 Months (*n* = 441)	*p*-Value ^2^
EAAs								
Histidine	28.2 (9.1)	28.0 (9.1)	29.7 (13.9)	0.944	1795.2 (264.3)	1803.3 (255.6)	1326.2 (201.4)	<0.001 ^b^
Isoleucine	16.5 (10.3)	15.9 (9.8)	12.7 (5.9)	<0.001 ^b^	4034.8 (599.5)	4094.8 (609.8)	3381.8 (461.0)	<0.001 ^b^
Leucine	33.9 (17.8)	32.8 (15.7)	34.9 (11.4)	<0.001 ^a^	8690.4 (1232.6)	8728.5 (1188.1)	6791.8 (922.6)	<0.001 ^b^
Lysine	34.9 (24.1)	32.3 (20.4)	30.2 (15.0)	0.333	6359.2 (949.1)	6371.2 (895.8)	3963.3 (626.2)	<0.001 ^b^
Methionine	27.8 (24.2)	29.5 (22.7)	34.6 (22.7)	<0.001 ^a^	1010.6 (200.7)	1019.2 (208.4)	750.4 (220.2)	<0.001 ^b^
Phenylalanine	21.4 (10.1)	21.3 (10.1)	16.5 (5.6)	<0.001 ^b^	2610.9 (422.4)	2594.4 (382.6)	2057.7 (305.0)	<0.001 ^b^
Threonine	71.0 (29.3)	70.8 (30.6)	97.1 (37.2)	<0.001 ^a^	3707.5 (578.2)	3688.9 (552.9)	3367.3 (507.9)	<0.001 ^b^
Tryptophan	14.6 (7.5)	14.5 (7.4)	29.0 (11.8)	<0.001 ^a^	-	-	-	-
Valine	109.7 (58.2)	110.5 (54.9)	66.7 (27.4)	<0.001 ^b^	4669.9 (755.3)	4678.7 (740.3)	4198.7 (628.1)	<0.001 ^b^
NEAAs/conditionally EAAs								
Alanine	231.7 (64.8)	228.2 (62.6)	242.7 (72.2)	<0.001 ^a^	5354.5 (988.8)	5294.8 (864.6)	3974.9 (639.3)	<0.001 ^b^
Arginine	17.0 (11.3)	15.9 (9.5)	17.0 (6.4)	0.029	2924.9 (1048.9)	2925.1 (1006.3)	2141.1 (589.8)	<0.001 ^b^
Asparagine	24.9 (14.9)	25.7 (14.8)	17.7 (8.6)	<0.001 ^b^	8558.3 (1324.2) ^3^	8527.1 (1221.1) ^3^	6240.4 (932.3) ^3^	<0.001 ^b^
Aspartate	30.9 (18.2)	30.0 (17.4)	65.3 (39.1)	<0.001 ^a^				
Glutamate	1307.0 (410.3)	1270.8 (395.3)	1530.1 (328.5)	<0.001 ^a^	13,477.7 (1630.0) ^4^	13,533.9 (1589.8) ^4^	11,478.3 (1550.2) ^4^	<0.001 ^b^
Glutamine	239.0 (161.0)	249.9 (162.9)	548.5 (268.6)	<0.001 ^a^				
Glycine	139.1 (40.4)	138.7 (37.1)	146.3 (48.5)	0.056	3902.8 (709.3)	3837.5 (583.5)	2868.4 (516.4)	<0.001 ^b^
Serine	100.2 (34.6)	100.0 (33.1)	137.8 (61.7)	<0.001 ^a^	5120.4 (817.2)	5068.5 (719.1)	4094.4 (626.5)	<0.001 ^b^
Tyrosine	27.5 (20.1)	28.0 (21.0)	17.5 (7.8)	<0.001 ^b^	1897.6 (296.9)	1896.1 (281.8)	1506.3 (224.8)	<0.001 ^b^
Non-coded AAs								
Citrulline	10.7 (4.7)	10.7 (5.0)	20.8 (6.1)	<0.001 ^a^	-	-	-	-
Ornithine	14.8 (16.7)	14.1 (15.1)	15.7 (27.0)	<0.001 ^a^	-	-	-	-
Taurine	243.6 (93.1)	240.9 (94.3)	266.2 (106.1)	<0.001 ^a^	-	-	-	-
Sum								
All AAs	2744.5 (645.1)	2708.5 (627.4)	3377.1 (676.4)	<0.001 ^a^	74,114.8 (11,817.4)	74,062.2 (11,099.8)	58,140.9 (8951.5)	<0.001 ^b^

AA: amino acid; FAA: free amino acid; TAA: total amino acid; EAA: essential amino acid; NEAA: non-essential amino acid. Data are expressed as µmol/L and reported as mean (SD). Significant differences, assessed by Wilcoxon rank-sum tests, between amino acid levels at 6 weeks and at 6 months are defined as follows: ^a^ Increase from 6 weeks to 6 months; ^b^ decrease from 6 weeks to 6 months. For this comparison, the 6-month data was restricted to mothers who also provided a sample at 6 weeks (“6 weeks restricted”). ^1^ Bonferroni-adjusted level of statistical significance is α = 0.05/22 = 0.002; ^2^ Bonferroni adjusted level of statistical significance is α = 0.05/16 = 0.003; ^3^ asparagine + aspartate; ^4^ glutamate + glutamine.

## Data Availability

Due to participant consent and data protection, we may not be able to share the raw data. However, the authors are open to sharing aggregate data, which have been included as Supplementary Materials.

## Supplementary Materials

The following are available online at https://www.mdpi.com/article/10.3390/nu13062009/s1, Figure S1: Compositional biplots from principal component analysis of free amino acid levels in 6-week and 6-month human milk, Table S1: Free amino acid patterns in 6-week human milk obtained from factor analyses, Table S2A: Levels of free amino acids in human milk for boys and girls, Table S2B: Levels of total amino acids in human milk for boys and girls, Table S3A: Spearman correlation coefficients between free amino acids in human milk and maternal anthropometrics, Table S3B: Spearman correlation coefficients between total amino acids in human milk and maternal anthropometrics, Table S3C: Spearman correlation coefficients between free amino acids in human milk and infant anthropometrics, Table S3D: Spearman correlation coefficients between total amino acids in human milk and infant anthropometrics, Table S4: Partial correlation coefficients between amino acids in human milk and infant and maternal factors, Table S5A: Free amino acid levels in human milk of mothers with or without an allergy, Table S5B: Total amino acid levels in human milk of mothers with or without an allergy, Table S6A: Adjusted model results for associations between free amino acids in human milk and infant wheeze outcomes, Table S6B: Adjusted model results for associations of free amino acids in human milk and infant atopic disease and lower respiratory tract infections, Table S6C: Adjusted model results for associations between free amino acids in human milk with infant food allergy.
