# Supplementary material for: Free and Total Amino Acids in Human Milk in Relation to Maternal and Infant Characteristics and Infant Health Outcomes: The Ulm SPATZ Health Study
Source: Nutrients. 2021 Jun 10;13(6):2009. doi: 10.3390/nu13062009 (PMC8230437; doi:10.3390/nu13062009)
Supplement: Supplementary file 1 [file nutrients-13-02009-s001.zip › nutrients-1225337-supplementary.pdf]

Supplementary Materials

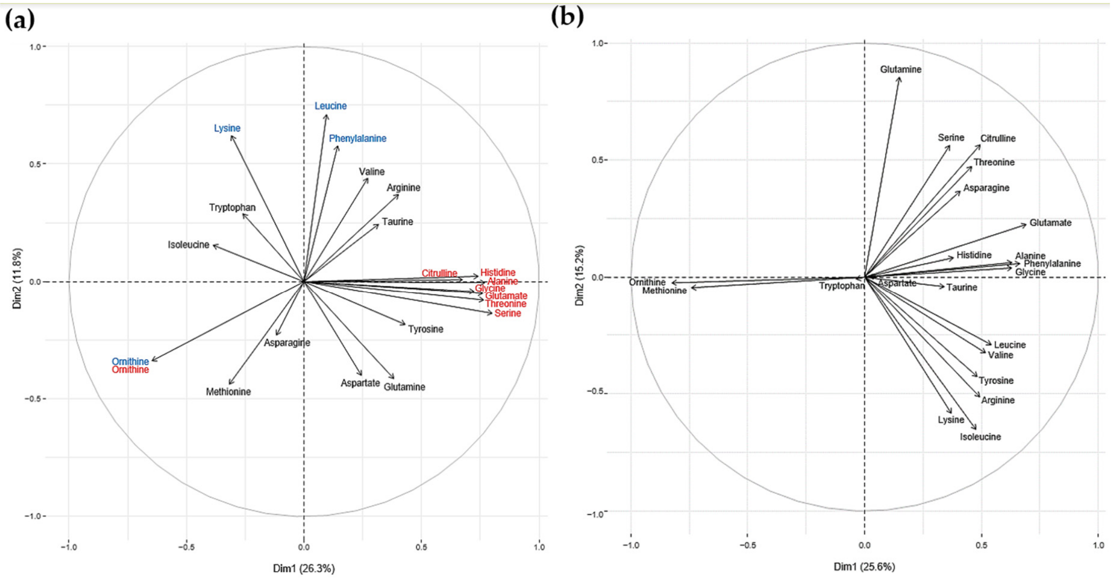

Figure S1. Compositional biplots from principal component analysis of free amino acid (FAA) levels in 6-week human milk (HM) (**a**) and 6-month HM (**b**). Potential patterns of FAAs as revealed by factor analyses are colored (red or blue).

Table S1. Free amino acid patterns in 6-week human milk obtained from factor analyses.

FAA	Factors loadings	
	Factor 1	Factor 2
**EAAs**		
**Histidine**	0.64	-
**Isoleucine**	-	-
**Leucine**	-	0.75
**Lysine**	-	0.51
**Methionine**	-	-
**Phenylalanine**	-	0.59
**Threonine**	0.76	-
**Tryptophan**	-	-
**Valine**	-	-
**NEAAs/conditionally EAAs**		
**Alanine**	0.66	-
**Arginine**	-	-
**Asparagine**	-	-
**Aspartate**	-	-

**Glutamate**	0.68	-
**Glutamine**	-	-
**Glycine**	0.66	-
**Serine**	0.83	-
**Tyrosine**	-	-
**Non-coded AAs**		
**Citrulline**	0.58	**-**
**Ornithine**	-0.54	-0.56
**Taurine**	-	**-**

FAA: free amino acid; EAA: essential amino acid; NEAA: non-essential amino acid; AA: amino acid; HM: human milk. Values are factor loadings from principal component analysis. Only loadings < -0.50 and > 0.50 are shown.

Table S2A. Levels of free amino acids in human milk for boys and girls.

**FAA (μmol/L)**	**6 weeks Mean (SD)**		**6 months Mean (SD)**		**Analysis of differences at 6 weeks (*p*-value)**	**Analysis of differences at 6 months (*p*-value)**
	**HM for boys (n = 354)**	**HM for girls (n = 317)**	**HM for boys (n = 232)**	**HM for girls (n = 209)**		
**EAAs**						
**Histidine**	28.4 (9.3)	28.1 (8.9)	29.6 (14.6)	29.9 (13.2)	0.661	0.397
**Isoleucine**	16.7 (10.5)	16.2 (10.1)	12.6 (6.4)	12.9 (5.3)	0.624	0.136
**Leucine**	33.7 (16.6)	34.2 (19.1)	34.0 (12.0)	35.8 (10.8)	0.930	0.038
**Lysine**	35.1 (25.2)	34.6 (22.8)	29.9 (16.3)	30.5 (13.6)	0.708	0.104
**Methionine**	27.7 (23.9)	28.0 (24.6)	33.8 (26.9)	35.5 (27.6)	0.875	0.588
**Phenylalanine**	21.7 (10.0)	21.0 (10.2)	16.5 (6.1)	16.4 (5.0)	0.283	0.829
**Threonine**	71.2 (29.7)	70.9 (29.0)	94.8 (38.0)	99.6 (36.3)	0.863	0.081
**Tryptophan**	14.3 (7.4)	14.9 (7.6)	28.6 (11.8)	29.5 (11.8)	0.381	0.227
**Valine**	110.4 (58.1)	109.0 (58.4)	65.4 (26.0)	68.1 (28.9)	0.710	0.277
**NEAAs/conditionally EAAs**						
**Alanine**	233.8 (65.1)	229.3 (64.5)	241.2 (75.0)	244.5 (69.0)	0.348	0.391
**Arginine**	17.0 (12.0)	17.1 (10.5)	16.9 (6.9)	17.2 (5.8)	0.468	0.169
**Asparagine**	25.6 (14.9)	24.3 (15.0)	17.4 (7.7)	18.0 (9.5)	0.153	0.998
**Aspartate**	31.1 (18.0)	30.6 (18.4)	63.8 (37.3)	66.9 (40.9)	0.551	0.537
**Glutamate**	1326.8 (415.7)	1284.9 (403.7)	1498.3 (320.6)	1565.3 (334.3)	0.196	0.023
**Glutamine**	244.0 (160.2)	233.5 (162.0)	547.9 (258.4)	549.2 (280.0)	0.244	0.713
**Glycine**	138.9 (41.3)	139.3 (39.5)	143.8 (49.9)	149.1 (47.0)	0.792	0.072

**Serine**	101.6 (34.0)	98.6 (35.3)	135.4 (62.7)	140.5 (60.7)	0.224	0.207
**Tyrosine**	27.9 (20.2)	27.0 (20.2)	17.7 (8.3)	17.2 (7.3)	0.297	0.760
**Non-coded AAs**						
**Citrulline**	11.0 (4.9)	10.4 (4.4)	20.9 (5.8)	20.6 (6.3)	0.199	0.300
**Ornithine**	15.1 (17.9)	14.4 (15.3)	15.5 (29.8)	16.0 (23.4)	0.563	0.176
**Taurine**	233.2 (90.3)	255.2 (95.0)	260.8 (108.3)	272.3 (103.7)	**< 0.001***	0.076
**Sum**						
**All FAAs**	2765.0 (642.1)	2721.5 (648.6)	3324.6 (652.9)	3435.1 (698.4)	0.353	0.126

FAA: free amino acid; EAA: essential amino acid; NEAA: non-essential amino acid; AA: amino acid; HM: human milk. * p-value remains statistically significant following Bonferroni adjustment. Bonferroni-adjusted level of statistical significance is $\alpha = 0.05/22 = 0.002$. P-values < 0.002 are indicated in bold.

Table S2B. Levels of total amino acids in human milk for boys and girls.

TAA (μmol/L)	6 weeks Mean (SD)		6 months Mean (SD)		Analysis of differences at 6 weeks (p-value)	Analysis of differences at 6 months (p-value)
	HM for boys (n = 354)	HM for girls (n = 317)	HM for boys (n = 232)	HM for girls (n = 209)		
**EAAs**						
**Histidine**	1818.4 (279.1)	1769.3 (244.7)	1317.0 (191.0)	1336.3 (212.4)	0.054	0.592
**Isoleucine**	4087.7 (637.6)	4087.7 (549.0)	3365.5 (443.1)	3399.8 (480.4)	0.833	0.463
**Leucine**	8796.3 (1309.8)	8572.2 (1130.6)	6761.9 (889.3)	6824.8 (959.1)	0.056	0.563
**Lysine**	6411.4 (983.4)	6301.1 (907.3)	3931.2 (584.5)	3998.8 (668.7)	0.206	0.517
**Methionine**	1019.0 (217.1)	1001.2 (180.7)	742.1 (133.4)	759.6 (287.1)	0.455	0.831
**Phenylalanine**	2639.3 (449.1)	2579.2 (388.7)	2044.7 (292.6)	2072.0 (318.2)	0.140	0.446
**Threonine**	3726.5 (579.8)	3686.2 (576.6)	3339.1 (487.3)	3398.6 (529.1)	0.371	0.307
**Valine**	4732.5 (794.9)	4600.1 (703.2)	4176.7 (621.3)	4223.0 (636.2)	0.050	0.334
**NEAAs/conditionally EAAs**						
**Alanine**	5381.9 (980.8)	5323.9 (998.2)	3954.1 (628.6)	3997.8 (651.5)	0.525	0.561
**Arginine**	2932.3 (1054.6)	2916.6 (1044.0)	2124.3 (559.5)	2159.6 (622.4)	0.879	0.938
**Asx**	8629.1 (1366.4)	8479.1 (1272.9)	6192.6 (917.7)	6293.0 (947.6)	0.237	0.431
**Glx**	13667.2 (1730.6)	13266.1 (1484.2)	11387.5 (1603.9)	11578.4 (1485.9)	0.007‡	0.405
**Glycine**	3904.4 (695.2)	3901.1 (725.7)	2842.6 (505.1)	2896.8 (528.2)	0.945	0.336
**Serine**	5141.9 (814.7)	5096.2 (820.6)	4064.3 (605.2)	4127.5 (648.8)	0.517	0.367
**Tyrosine**	1916.1 (307.8)	1877.0 (283.2)	1502.6 (220.6)	1510.4 (229.8)	0.187	0.783
**Sum**						

**All TAAs (mmol/L)**	74.8 (12.2)	73.5 (11.3)	57.7 (8.7)	58.6 (9.2)	0.129	0.435

TAA: total amino acid; Glx: glutamate + glutamine; Asx: aspartate + asparagine; HM: human milk. ‡ $p < 0.01$. Bonferroni-adjusted level of statistical significance is $\alpha = 0.05/16 = 0.003$.

Table S3A. Spearman correlation coefficients between free amino acids in human milk and maternal anthropometrics.

FAA	Maternal anthropometrics							
	Age		Weight		Height		BMI	
	6 weeks Rho (95% CI)	6 months Rho (95% CI)	6 weeks Rho (95% CI)	6 months Rho (95% CI)	6 weeks Rho (95% CI)	6 months Rho (95% CI)	6 weeks Rho (95% CI)	6 months Rho (95% CI)
**EAAs**								
**Histidine**	-0.02 (-0.09; 0.06)	0.00 (-0.09; 0.10)	-0.03 (-0.10; 0.05)	-0.04 (-0.13; 0.06)	0.02 (-0.06; 0.09)	-0.02 (-0.11; 0.07)	-0.03 (-0.11; 0.04)	-0.03 (-0.12; 0.07)
**Isoleucine**	-0.11 (-0.18; -0.03)‡	-0.02 (-0.11; 0.08)	0.01 (-0.07; 0.09)	0.09 (-0.01; 0.18)	-0.05 (-0.12; 0.03)	-0.05 (-0.14; 0.05)	0.08 (-0.01; 0.16)	0.12 (0.03; 0.21)‡
**Leucine**	-0.04 (-0.12; 0.03)	0.01 (-0.08; 0.10)	0.03 (-0.04; 0.11)	0.06 (-0.04; 0.15)	-0.09 (-0.17; -0.02)	-0.01 (-0.10; 0.09)	0.11 (0.04; 0.19)‡	0.09 (-0.01; 0.18)
**Lysine**	-0.03 (-0.11; 0.05)	0.06 (-0.04; 0.15)	-0.04 (-0.12; 0.03)	0.06 (-0.03; 0.15)	-0.11 (-0.18; -0.04)‡	-0.02 (-0.12; 0.07)	0.01 (-0.07; 0.09)	0.05 (-0.03; 0.13)
**Methionine**	0.02 (-0.06; 0.09)	0.09 (0.00; 0.19)	-0.03 (-0.10; 0.05)	0.00 (-0.09; 0.10)	-0.03 (-0.11; 0.04)	0.00 (-0.09; 0.10)	-0.01 (-0.09; 0.07)	0.01 (-0.08; 0.11)
**Phenylalanine**	0.03 (-0.04; 0.11)	0.06 (-0.03; 0.15)	0.03 (-0.05; 0.10)	-0.06 (-0.15; 0.04)	-0.05 (-0.12; 0.03)	0.04 (-0.05; 0.14)	0.05 (-0.02; 0.13)	-0.07 (-0.16; 0.02)
**Threonine**	0.01 (-0.07; 0.08)	0.14 (0.04; 0.23)‡	-0.05 (-0.13; 0.02)	-0.11 (-0.20; -0.01)	-0.01 (-0.09; 0.07)	0.02 (-0.08; 0.11)	-0.06 (-0.13; 0.02)	-0.12 (-0.21; -0.03)
**Tryptophan**	0.07 (-0.01; 0.15)	**0.16 (0.07; 0.25)***	0.01 (-0.07; 0.08)	0.03 (-0.07; 0.12)	0.05 (-0.03; 0.12)	0.01 (-0.08; 0.10)	0.01 (-0.07; 0.08)	0.02 (-0.08; 0.11)
**Valine**	0.03 (-0.05; 0.10)	0.06 (-0.04; 0.15)	0.03 (-0.04; 0.10)	0.05 (-0.02; 0.12)	0.00 (-0.08; 0.07)	-0.07 (-0.16; 0.03)	0.08 (-0.01; 0.17)	0.09 (-0.02; 0.19)
**NEAAs/Conditionally EAAs**								
**Alanine**	-0.03 (-0.11; 0.04)	0.04 (-0.06; 0.13)	0.04 (-0.04; 0.12)	0.07 (-0.02; 0.16)	-0.06 (-0.13; 0.02)	-0.05 (-0.14; 0.04)	0.07 (-0.01; 0.14)	0.08 (-0.02; 0.17)
**Arginine**	-0.08 (-0.16; -0.01)	0.04 (-0.06; 0.13)	0.02 (-0.06; 0.09)	0.13 (0.03; 0.22)‡	-0.09 (-0.16; -0.01)	-0.10 (-0.19; 0.00)	0.07 (-0.01; 0.14)	0.16 (0.06; 0.24)‡
**Asparagine**	0.00 (-0.08; 0.07)	0.04 (-0.06; 0.13)	**-0.12 (-0.20; -0.05)***	**-0.28 (-0.36; -0.19)***	0.01 (-0.07; 0.08)	0.03 (-0.06; 0.13)	**-0.14 (-0.22; -0.07)***	**-0.33 (-0.41; -0.24)***
**Aspartate**	0.00 (-0.08; 0.07)	-0.01 (-0.08; 0.07)	-0.05 (-0.12; 0.02)	-0.06 (-0.14; 0.02)	0.05 (-0.01; 0.11)	0.06 (0.00; 0.12)	-0.08 (-0.17; 0.02)	-0.10 (-0.20; 0.01)
**Glutamate**	0.01 (-0.07; 0.08)	0.00 (-0.09; 0.10)	-0.04 (-0.10; 0.02)	-0.05 (-0.12; 0.02)	0.01 (-0.04; 0.06)	0.04 (-0.03; 0.11)	-0.05 (-0.12; 0.03)	-0.08 (-0.18; 0.02)
**Glutamine**	0.05 (-0.03; 0.13)	0.06 (-0.04; 0.15)	**-0.12 (-0.19; -0.04)***	**-0.18 (-0.27; -0.09)***	0.04 (-0.03; 0.12)	0.09 (-0.01; 0.18)	**-0.15 (-0.22; -0.08)***	**-0.24 (-0.32; -0.15)***
**Glycine**	-0.06 (-0.14; 0.01)	0.06 (-0.04; 0.15)	-0.11 (-0.19; -0.04)‡	-0.02 (-0.11; 0.08)	-0.01 (-0.09; 0.06)	0.03 (-0.06; 0.12)	**-0.13 (-0.20; -0.05)***	-0.05 (-0.14; 0.05)
**Serine**	-0.03 (-0.11; 0.04)	0.07 (-0.03; 0.16)	-0.11 (-0.19; -0.04)‡	-0.12 (-0.21; -0.03)‡	-0.01 (-0.09; 0.06)	-0.03 (-0.12; 0.07)	**-0.13 (-0.20; -0.06)***	-0.13 (-0.22; -0.04)‡
**Tyrosine**	-0.02 (-0.09; 0.06)	0.07 (-0.02; 0.17)	0.03 (-0.05; 0.11)	0.12 (0.03; 0.21)	-0.03 (-0.10; 0.05)	-0.01 (-0.1; 0.08)	0.04 (-0.03; 0.12)	0.14 (0.05; 0.23)‡
**Non-coded AAs**								
**Citrulline**	0.03 (-0.04; 0.11)	0.13 (0.03; 0.22)‡	-0.02 (-0.09; 0.06)	-0.07 (-0.16; 0.02)	0.03 (-0.05; 0.10)	0.04 (-0.06; 0.13)	-0.03 (-0.11; 0.04)	-0.11 (-0.20; -0.01)
**Ornithine**	0.02 (-0.06; 0.09)	0.01 (-0.08; 0.11)	-0.02 (-0.09; 0.06)	-0.01 (-0.11; 0.08)	0.04 (-0.03; 0.12)	-0.05 (-0.14; 0.04)	-0.04 (-0.12; 0.03)	0.01 (-0.09; 0.10)
**Taurine**	0.05 (-0.02; 0.13)	0.11 (0.02; 0.20)	0.06 (-0.01; 0.14)	0.05 (-0.05; 0.14)	0.06 (-0.02; 0.14)	0.03 (-0.07; 0.12)	0.05 (-0.02; 0.13)	0.04 (-0.06; 0.13)
**Sum**								
**All FAAs**	0.01 (-0.06; 0.09)	0.05 (-0.05; 0.14)	-0.05 (-0.12; 0.03)	-0.07 (-0.17; 0.03)	-0.02 (-0.08; 0.05)	0.03 (-0.05; 0.10)	-0.06 (-0.14; 0.03)	-0.09 (-0.19; 0.02)

FAA: free amino acid; EAA: essential amino acid; NEAA: non-essential amino acid; BMI: body mass index. ‡ $p < 0.01$, * p-value remains statistically significant following Bonferroni adjustment. Bonferroni-adjusted level of statistical significance is $\alpha = 0.05/22 = 0.002$. P-values < 0.002 are indicated in bold.

Table S3B. Spearman correlation coefficients between total amino acids in human milk and maternal anthropometrics.

TAA	Maternal anthropometrics							
	Age		Weight		Height		BMI	
	6 weeks Rho (95% CI)	6 months Rho (95% CI)	6 weeks Rho (95% CI)	6 months Rho (95% CI)	6 weeks Rho (95% CI)	6 months Rho (95% CI)	6 weeks Rho (95% CI)	6 months Rho (95% CI)
**EAAs**								
**Histidine**	0.02 (-0.06; 0.09)	0.11 (0.02; 0.20)	0.02 (-0.05; 0.10)	0.13 (0.04; 0.22)‡	0.04 (-0.04; 0.11)	-0.06 (-0.15; 0.04)	0.00 (-0.08; 0.07)	0.13 (0.04; 0.22)‡
**Isoleucine**	0.08 (0.01; 0.16)	0.12 (0.02; 0.21)	0.02 (-0.05; 0.10)	0.12 (0.03; 0.21)	0.06 (-0.02; 0.14)	-0.02 (-0.11; 0.07)	0.00 (-0.08; 0.07)	0.10 (0.00; 0.19)
**Leucine**	0.05 (-0.02; 0.13)	0.12 (0.02; 0.21)	0.02 (-0.06; 0.10)	**0.15 (0.06; 0.24)***	0.04 (-0.03; 0.12)	-0.03 (-0.13; 0.06)	0.00 (-0.08; 0.08)	**0.14 (0.05; 0.23)***
**Lysine**	0.04 (-0.04; 0.12)	0.12 (0.02; 0.21)	-0.02 (-0.09; 0.06)	0.11 (0.02; 0.21)	0.02 (-0.05; 0.10)	-0.04 (-0.14; 0.05)	-0.03 (-0.11; 0.04)	0.11 (0.02; 0.20)
**Methionine**	0.03 (-0.05; 0.11)	0.14 (0.04; 0.23)‡	0.09 (0.01; 0.17)	0.09 (0.00; 0.18)	0.06 (-0.02; 0.13)	0.00 (-0.09; 0.09)	0.06 (-0.01; 0.14)	0.07 (-0.02; 0.16)
**Phenylalanine**	0.00 (-0.07; 0.08)	0.10 (0.01; 0.19)	0.04 (-0.03; 0.12)	**0.17 (0.08; 0.26)***	0.03 (-0.05; 0.10)	-0.04 (-0.13; 0.06)	0.03 (-0.05; 0.10)	**0.17 (0.07; 0.26)***
**Threonine**	0.02 (-0.06; 0.10)	0.10 (0.01; 0.19)	0.07 (-0.01; 0.14)	**0.16 (0.07; 0.25)***	0.00 (-0.07; 0.08)	-0.05 (-0.15; 0.04)	0.07 (-0.01; 0.15)	**0.16 (0.07; 0.25)***
**Valine**	0.03 (-0.05; 0.10)	0.10 (0.01; 0.19)	0.09 (0.01; 0.16)	**0.19 (0.10; 0.28)***	0.02 (-0.06; 0.09)	-0.02 (-0.11; 0.08)	0.08 (0.00; 0.15)‡	**0.18 (0.09; 0.27)***
**NEAAs/conditionally EAAs**								
**Alanine**	0.00 (-0.07; 0.08)	0.06 (-0.03; 0.16)	-0.01 (-0.09; 0.06)	**0.18 (0.08; 0.27)***	-0.04 (-0.11; 0.04)	-0.05 (-0.15; 0.04)	-0.01 (-0.08; 0.07)	**0.18 (0.09; 0.27)***
**Arginine**	0.00 (-0.08; 0.08)	0.00 (-0.09; 0.10)	0.04 (-0.04; 0.12)	0.07 (-0.03; 0.16)	-0.02 (-0.09; 0.06)	-0.07 (-0.17; 0.02)	0.05 (-0.03; 0.12)	0.10 (0.00; 0.19)
**Asx**	0.02 (-0.06; 0.09)	0.09 (0.00; 0.19)	0.01 (-0.07; 0.08)	**0.16 (0.07; 0.25)***	0.01 (-0.06; 0.09)	-0.06 (-0.15; 0.03)	0.00 (-0.08; 0.07)	**0.16 (0.07; 0.25)***
**Glx**	0.06 (-0.01; 0.14)	0.12 (0.03; 0.21)	0.01 (-0.06; 0.09)	0.10 (0.00; 0.19)	0.05 (-0.03; 0.12)	-0.04 (-0.13; 0.06)	-0.01 (-0.09; 0.06)	0.08 (-0.01; 0.17)
**Glycine**	-0.01 (-0.08; 0.07)	0.08 (-0.02; 0.17)	0.02 (-0.06; 0.09)	**0.16 (0.07; 0.25)***	-0.01 (-0.09; 0.07)	-0.04 (-0.14; 0.05)	0.02 (-0.05; 0.10)	**0.16 (0.07; 0.25)***
**Serine**	0.01 (-0.07; 0.09)	0.09 (0.00; 0.18)	0.03 (-0.04; 0.11)	**0.17 (0.08; 0.26)***	0.01 (-0.07; 0.08)	-0.06 (-0.16; 0.03)	0.03 (-0.04; 0.11)	**0.18 (0.09; 0.27)***

**Tyrosine**	0.03 (-0.04; 0.11)	0.10 (0.00; 0.19)	0.04 (-0.04; 0.11)	**0.19 (0.10; 0.28)***	0.04 (-0.04; 0.11)	-0.02 (-0.11; 0.08)	0.02 (-0.06; 0.09)	**0.17 (0.08; 0.26)***
**Sum**								
**All TAAs**	0.03 (-0.04; 0.11)	0.11 (0.02; 0.20)	0.03 (-0.05; 0.11)	**0.15 (0.05; 0.24)***	0.03 (-0.05; 0.10)	-0.05 (-0.15; 0.04)	0.01 (-0.06; 0.09)	**0.14 (0.05; 0.23)***

TAA: total amino acid; EAA: essential amino acid; NEAA: non-essential amino acid; Glx: glutamate + glutamine; Asx: aspartate + asparagine; BMI: body mass index. ‡ $p < 0.01$, * *p*-value remains statistically significant following Bonferroni adjustment. Bonferroni-adjusted level of statistical significance is $\alpha = 0.05/16 = 0.003$. *P*-values < 0.003 are indicated in bold.

Table S3C. Spearman correlation coefficients between free amino acids in human milk and infant anthropometrics.

FAA	Infant anthropometrics							
	Birth length		Birth weight		Gain in length		Gain in weight	
	6 weeks Rho (95% CI)	6 months Rho (95% CI)	6 weeks Rho (95% CI)	6 months Rho (95% CI)	6 weeks Rho (95% CI)	6 months Rho (95% CI)	6 weeks Rho (95% CI)	6 months Rho (95% CI)
**EAA**								
**Histidine**	0.00 (-0.08; 0.08)	-0.10 (-0.20; -0.01)	0.04 (-0.04; 0.12)	-0.02 (-0.12; 0.08)	0.01 (-0.07; 0.09)	0.02 (-0.08; 0.11)	0.08 (0.00; 0.16)	0.03 (-0.07; 0.12)
**Isoleucine**	-0.04 (-0.12; 0.04)	-0.02 (-0.11; 0.08)	-0.08 (-0.16; 0.00)	0.03 (-0.06; 0.13)	-0.04 (-0.12; 0.04)	0.01 (-0.09; 0.10)	-0.01 (-0.09; 0.07)	-0.01 (-0.1; 0.09)
**Leucine**	0.01 (-0.07; 0.08)	-0.04 (-0.13; 0.06)	-0.01 (-0.09; 0.07)	0.03 (-0.07; 0.12)	0.00 (-0.08; 0.08)	0.02 (-0.08; 0.12)	0.03 (-0.05; 0.11)	-0.01 (-0.11; 0.08)
**Lysine**	-0.03 (-0.11; 0.05)	-0.06 (-0.16; 0.03)	-0.01 (-0.09; 0.07)	-0.02 (-0.11; 0.08)	-0.07 (-0.15; 0.01)	0.04 (-0.06; 0.13)	-0.03 (-0.11; 0.05)	-0.01 (-0.11; 0.08)
**Methionine**	-0.02 (-0.10; 0.06)	-0.05 (-0.14; 0.05)	-0.07 (-0.14; 0.01)	-0.03 (-0.12; 0.07)	0.01 (-0.07; 0.09)	-0.03 (-0.12; 0.07)	-0.03 (-0.11; 0.05)	0.03 (-0.07; 0.12)
**Phenylalanine**	0.02 (-0.06; 0.10)	-0.05 (-0.15; 0.05)	0.01 (-0.07; 0.09)	0.02 (-0.08; 0.11)	-0.05 (-0.13; 0.03)	0.08 (-0.02; 0.17)	-0.04 (-0.12; 0.04)	0.08 (-0.01; 0.17)
**Threonine**	-0.03 (-0.11; 0.05)	-0.08 (-0.18; 0.01)	0.02 (-0.06; 0.10)	-0.02 (-0.11; 0.08)	0.04 (-0.04; 0.12)	0.00 (-0.10; 0.09)	0.11 (0.03; 0.19)‡	-0.02 (-0.12; 0.07)
**Tryptophan**	0.04 (-0.04; 0.12)	0.11 (0.01; 0.20)	-0.03 (-0.11; 0.05)	0.05 (-0.05; 0.14)	-0.05 (-0.13; 0.03)	0.04 (-0.06; 0.13)	0.03 (-0.05; 0.11)	0.01 (-0.08; 0.11)
**Valine**	0.00 (-0.08; 0.08)	-0.07 (-0.17; 0.02)	0.03 (-0.05; 0.11)	-0.04 (-0.14; 0.05)	-0.07 (-0.15; 0.01)	-0.02 (-0.11; 0.08)	-0.05 (-0.13; 0.03)	0.00 (-0.10; 0.09)
**NEAAs/conditionally EAAs**								
**Alanine**	0.00 (-0.08; 0.08)	-0.01 (-0.11; 0.08)	0.02 (-0.06; 0.09)	-0.03 (-0.12; 0.07)	0.02 (-0.06; 0.10)	0.00 (-0.10; 0.10)	0.03 (-0.05; 0.11)	-0.05 (-0.15; 0.04)
**Arginine**	-0.08 (-0.16; 0.00)	0.01 (-0.08; 0.11)	-0.07 (-0.14; 0.02)	-0.06 (-0.16; 0.03)	-0.02 (-0.10; 0.06)	-0.01 (-0.10; 0.09)	-0.02 (-0.10; 0.06)	-0.01 (-0.10; 0.09)
**Asparagine**	-0.01 (-0.09; 0.07)	-0.08 (-0.17; 0.02)	-0.06 (-0.14; 0.02)	-0.06 (-0.16; 0.03)	-0.02 (-0.10; 0.06)	0.03 (-0.06; 0.13)	0.06 (-0.02; 0.14)	0.01 (-0.09; 0.10)
**Aspartate**	0.05 (-0.03; 0.13)	0.01 (-0.08; 0.11)	-0.01 (-0.09; 0.07)	0.03 (-0.07; 0.12)	-0.09 (-0.17; -0.01)	-0.01 (-0.1; 0.09)	-0.02 (-0.10; 0.06)	-0.07 (-0.16; 0.03)
**Glutamate**	0.01 (-0.07; 0.09)	-0.02 (-0.12; 0.07)	0.02 (-0.06; 0.10)	0.01 (-0.09; 0.10)	0.00 (-0.08; 0.08)	-0.02 (-0.11; 0.08)	**0.12 (0.04; 0.20)***	-0.04 (-0.13; 0.06)

**Glutamine**	0.01 (-0.07; 0.09)	-0.03 (-0.12; 0.07)	0.00 (-0.08; 0.08)	0.00 (-0.10; 0.09)	0.10 (0.05; 0.15)‡	0.04 (-0.06; 0.13)	**0.22 (0.14; 0.29)***	0.06 (-0.03; 0.16)
**Glycine**	-0.01 (-0.09; 0.07)	-0.04 (-0.13; 0.06)	-0.01 (-0.09; 0.07)	0.01 (-0.08; 0.11)	0.01 (-0.07; 0.09)	0.06 (-0.03; 0.16)	0.05 (-0.03; 0.13)	0.03 (-0.06; 0.13)
**Serine**	0.02 (-0.07; 0.09)	-0.08 (-0.17; 0.02)	0.00 (-0.08; 0.08)	-0.03 (-0.13; 0.06)	0.06 (-0.03; 0.13)	0.08 (-0.02; 0.17)	**0.19 (0.11; 0.27)***	0.08 (-0.02; 0.17)
**Tyrosine**	0.02 (-0.06; 0.10)	-0.01 (-0.11; 0.08)	-0.01 (-0.08; 0.08)	0.08 (-0.02; 0.17)	-0.07 (-0.15; 0.01)	0.05 (-0.05; 0.15)	0.04 (-0.04; 0.12)	0.09 (-0.01; 0.18)
**Non-coded AAs**								
**Citrulline**	0.04 (-0.04; 0.12)	-0.05 (-0.15; 0.05)	0.02 (-0.06; 0.10)	-0.03 (-0.12; 0.07)	0.01 (-0.07; 0.09)	0.04 (-0.06; 0.13)	0.09 (0.01; 0.17)	0.02 (-0.08; 0.11)
**Ornithine**	0.00 (-0.08; 0.08)	-0.02 (-0.12; 0.07)	-0.03 (-0.11; 0.05)	-0.03 (-0.13; 0.06)	-0.09 (-0.17; -0.01)	0.01 (-0.09; 0.10)	-0.03 (-0.11; 0.05)	0.05 (-0.05; 0.14)
**Taurine**	0.07 (-0.01; 0.15)	0.09 (-0.01; 0.18)	0.04 (-0.04; 0.12)	0.08 (-0.02; 0.17)	0.06 (-0.02; 0.14)	0.04 (-0.05; 0.14)	0.05 (-0.03; 0.13)	-0.03 (-0.12; 0.07)
**Sum**								
**All FAAs**	0.02 (-0.06; 0.10)	-0.03 (-0.12; 0.07)	0.02 (-0.06; 0.10)	0.01 (-0.09; 0.10)	0.00 (-0.08; 0.08)	0.04 (-0.06; 0.13)	**0.16 (0.08; 0.24)***	0.00 (-0.09; 0.10)

FAA: free amino acid; EAA: essential amino acid; NEAA: non-essential amino acid. ‡ $p < 0.01$, * p-value remains statistically significant following Bonferroni adjustment. Bonferroni-adjusted level of statistical significance is $\alpha = 0.05/22 = 0.002$. P-values < 0.002 are indicated in bold.

Table S3D. Spearman correlation coefficients between total amino acids in human milk and infant anthropometrics.

TAA	Infant anthropometrics							
	Birth length		Birth weight		Gain in length		Gain in weight	
	6 weeks Rho (95% CI)	6 months Rho (95% CI)	6 weeks Rho (95% CI)	6 months Rho (95% CI)	6 weeks Rho (95% CI)	6 months Rho (95% CI)	6 weeks Rho (95% CI)	6 months Rho (95% CI)
**EAAs**								
**Histidine**	0.02 (-0.06; 0.10)	0.10 (0.01; 0.20)	-0.03 (-0.11; 0.05)	0.06 (-0.03; 0.16)	-0.11 (-0.19; -0.04)‡	-0.12 (-0.22; -0.03)	**-0.16 (-0.23; -0.08)***	-0.06 (-0.15; 0.04)
**Isoleucine**	-0.01 (-0.09; 0.07)	0.08 (-0.02; 0.17)	0.00 (-0.08; 0.08)	0.06 (-0.04; 0.15)	-0.06 (-0.13; 0.03)	-0.12 (-0.21; -0.03)	**-0.12 (-0.20; -0.04)***	-0.08 (-0.17; 0.02)
**Leucine**	0.03 (-0.05; 0.11)	0.10 (0.00; 0.19)	-0.01 (-0.09; 0.07)	0.07 (-0.02; 0.17)	-0.11 (-0.19; -0.03)‡	-0.13 (-0.22; -0.03)‡	**-0.12 (-0.20; -0.04)***	-0.07 (-0.16; 0.03)
**Lysine**	0.04 (-0.04; 0.12)	0.10 (0.01; 0.19)	-0.01 (-0.09; 0.07)	0.07 (-0.02; 0.17)	-0.12 (-0.2; -0.04)‡	-0.12 (-0.22; -0.03)	**-0.13 (-0.20; -0.05)***	-0.07 (-0.16; 0.03)
**Methionine**	-0.01 (-0.08; 0.07)	0.03 (-0.06; 0.13)	-0.03 (-0.11; 0.05)	0.03 (-0.07; 0.12)	-0.03 (-0.11; 0.05)	-0.08 (-0.18; 0.01)	-0.09 (-0.17; -0.01)	-0.05 (-0.14; 0.05)
**Phenylalanine**	0.02 (-0.06; 0.10)	0.09 (0.00; 0.18)	-0.03 (-0.11; 0.05)	0.07 (-0.02; 0.17)	-0.12 (-0.20; -0.04)‡	-0.11 (-0.20; -0.01)	**-0.16 (-0.23; -0.08)***	-0.05 (-0.15; 0.04)
**Threonine**	0.00 (-0.08; 0.08)	0.09 (-0.01; 0.18)	-0.01 (-0.09; 0.07)	0.06 (-0.03; 0.16)	-0.10 (-0.18; -0.02)	-0.11 (-0.20; -0.01)	**-0.16 (-0.23; -0.08)***	-0.06 (-0.16; 0.04)
**Valine**	0.00 (-0.08; 0.08)	0.04 (-0.06; 0.13)	-0.01 (-0.08; 0.08)	0.05 (-0.05; 0.14)	-0.10 (-0.18; -0.02)	-0.09 (-0.18; 0.00)	**-0.16 (-0.24; -0.08)***	-0.04 (-0.14; 0.05)
**NEAAs/conditionally EAAs**								
**Alanine**	0.05 (-0.03; 0.13)	0.09 (0.00; 0.19)	0.00 (-0.08; 0.08)	0.06 (-0.04; 0.15)	**-0.13 (-0.21; -0.05)***	-0.09 (-0.18; 0.01)	**-0.13 (-0.21; -0.06)***	-0.06 (-0.15; 0.04)
**Arginine**	0.03 (-0.05; 0.11)	0.11 (0.01; 0.20)	0.01 (-0.07; 0.09)	0.04 (-0.05; 0.14)	**-0.13 (-0.21; -0.06)***	-0.09 (-0.18; 0.01)	-0.12 (-0.20; -0.04)‡	-0.08 (-0.17; 0.02)
**Asx**	0.03 (-0.05; 0.11)	0.11 (0.01; 0.20)	-0.02 (-0.10; 0.06)	0.07 (-0.03; 0.16)	**-0.13 (-0.21; -0.05)***	-0.12 (-0.21; -0.02)	**-0.15 (-0.23; -0.07)***	-0.08 (-0.17; 0.02)
**Glx**	0.02 (-0.06; 0.10)	0.08 (-0.02; 0.17)	-0.01 (-0.09; 0.07)	0.06 (-0.04; 0.15)	-0.09 (-0.17; -0.01)	-0.13 (-0.22; -0.03)‡	-0.08 (-0.16; 0.00)	-0.09 (-0.18; 0.01)
**Glycine**	0.03 (-0.05; 0.11)	0.10 (0.00; 0.19)	-0.02 (-0.10; 0.06)	0.07 (-0.03; 0.16)	**-0.14 (-0.21; -0.06)***	-0.08 (-0.18; 0.01)	**-0.14 (-0.22; -0.06)***	-0.05 (-0.14; 0.05)
**Serine**	0.01 (-0.07; 0.09)	0.10 (0.00; 0.19)	-0.02 (-0.10; 0.06)	0.07 (-0.03; 0.16)	-0.12 (-0.20; -0.04)‡	-0.09 (-0.19; 0.00)	**-0.15 (-0.22; -0.07)***	-0.04 (-0.14; 0.05)

**Tyrosine**	0.02 (-0.06; 0.10)	0.13 (0.03; 0.22)	-0.01 (-0.09; 0.07)	0.10 (0.00; 0.19)	-0.10 (-0.18; -0.02)	-0.13 (-0.22; -0.03)‡	**-0.14 (-0.22; -0.06)***	-0.06 (-0.15; 0.04)
**Sum**								
**Sum of all TAAs**	0.03 (-0.06; 0.10)	0.10 (0.01; 0.19)	-0.02 (-0.10; 0.06)	0.07 (-0.03; 0.16)	**-0.13 (-0.21; -0.05)***	-0.12 (-0.22; -0.03)	**-0.15 (-0.23; -0.07)***	-0.08 (-0.17; 0.02)

TAA: total amino acid; EAA: essential amino acid; NEAA: non-essential amino acid; Glx: glutamate + glutamine; Asx: aspartate + asparagine. ‡ $p < 0.01$, * p-value remains statistically significant following Bonferroni adjustment. Bonferroni-adjusted level of statistical significance is $\alpha = 0.05/16 = 0.003$. P-values < 0.003 are indicated in bold.

Table S4. Partial correlation coefficients between amino acids in human milk and infant and maternal factors.

Anthropometric measurement	Sample collection time	Amino acid	Beta estimate (95% CI)	*p*-value	Putative confounders included
**Maternal characteristics**					
**Pre-pregnancy weight**	6 weeks	Free glutamine	-0.676 (-1.556; 0.205)	0.132	Infant gender, maternal age, history in allergic disease
		Free asparagine	-0.089 (-0.171; -0.008)	0.031†	
	6 months	Free glutamine	-2.686 (6.783; -1.410)	0.198	
		Free asparagine	-0.029 (-0.160; 0.102)	0.667	
		Sum of all TAAs	8.340 (-7.430; 24.111)	0.299	
**Pre-pregnancy BMI**	6 weeks	Free glutamine	-1.301 (-3.752; 1.150)	0.298	Infant gender, maternal age, history in allergic disease
		Free asparagine	-0.167 (-0.396; 0.063)	0.155	
		Free serine	-0.403 (-0.935; 0.128)	0.136	
		Free glycine	-0.513 (-1.135; 0.103)	0.102	
	6 months	Free glutamine	-0.916 (-6.101; 4.270)	0.729	
		Free asparagine	-0.099 (-0.268; 0.069)	0.245	
		Sum of all TAAs	9.938 (-10.177; 30.053)	0.332	
**Age**	6 months	Free tryptophan	0.298 (0.022; 0.574)	0.034†	Infant gender, maternal pre-pregnancy BMI, history in allergic disease
**Infant characteristics**					
**Gain in weight**	6 weeks	Free glutamate	0.032 (-0.016; 0.080)	0.190	Infant gender, maternal age, maternal pre-pregnancy BMI, history in allergic disease
		Free glutamine	0.015 (0.008; 0.023)	0.005‡	
		Free serine	0.004 (0.001; 0.008)	0.046†	
		Sum of all TAAs	-0.212 (-0.364; -0.060)	**0.003***	
	6 months	Sum of all TAAs	-0.155 (-0.267; -0.043)	0.007‡	
**Gain in length**	6 weeks	Free glutamine	5.436 (-0.811; 11.683)	0.088	
		Sum of all TAAs	-8.568 (-13.554; -3.582)	**<0.001***	

TAAs: total amino acids; BMI: body mass index. † $p < 0.05$, ‡ $p < 0.01$, * p-value remains statistically significant following Bonferroni adjustment. Bonferroni-adjusted level of statistical significance for FAAs is $\alpha = 0.05/16 = 0.002$ and for TAAs is $\alpha = 0.05/16 = 0.003$.

Table S5A. Free amino acid levels in human milk of mothers with or without an allergy.

FAA (µmol/L)	6 weeks Mean (SD)		6 months Mean (SD)		Analysis of differences at 6 weeks (*p*-value)	Analysis of differences at 6 months (*p*-value)
	Mothers without an allergy (n = 443)	Mothers with an allergy (n = 225)	Mothers without an allergy (n = 274)	Mothers with an allergy (n = 166)		
**EAAs**						
**Histidine**	28.1 (9.3)	28.6 (8.8)	28.6 (12.1)	31.2 (16.0)	0.456	0.068
**Isoleucine**	16.8 (10.8)	15.8 (9.1)	12.1 (5.1)	13.6 (6.8)	0.179	0.017
**Leucine**	34.1 (16.7)	33.6 (19.9)	34.0 (10.8)	36.3 (12.3)	0.740	0.045
**Lysine**	36.0 (25.6)	32.5 (20.6)	28.9 (12.9)	32.1 (17.6)	0.058	0.043
**Methionine**	27.9 (23.8)	27.9 (25.2)	33.1 (26.7)	36.8 (27.8)	0.997	0.162
**Phenylalanine**	21.3 (9.9)	21.3 (10.6)	16.2 (5.2)	16.8 (6.1)	0.972	0.325
**Threonine**	72.2 (30.7)	69.0 (26.4)	97.6 (38.8)	96.0 (34.7)	0.160	0.644
**Tryptophan**	14.4 (7.3)	14.9 (7.8)	29.3 (11.9)	28.7 (11.7)	0.444	0.613
**Valine**	108.6 (59.6)	111.4 (55.7)	65.2 (27.4)	69.0 (27.4)	0.546	0.157
**NEAAs/Conditionally EAAs**						
**Alanine**	231.9 (65.1)	230.4 (62.4)	238.4 (70.3)	250.0 (75.0)	0.780	0.108
**Arginine**	16.9 (12.0)	17.2 (10.0)	16.6 (5.8)	17.7 (7.3)	0.755	0.091
**Asparagine**	24.1 (14.4)	26.8 (15.8)	18.1 (8.6)	16.9 (8.6)	0.130	0.146
**Aspartate**	31.1 (18.0)	30.6 (18.6)	62.5 (36.5)	69.6 (42.7)	0.709	0.076
**Glutamate**	1325.2 (418.8)	1273.5 (391.3)	1531.4 (323.1)	1526.6 (338.6)	0.116	0.882
**Glutamine**	238.4 (160.6)	241.3 (162.4)	579.4 (268.0)	498.3 (263.4)	0.828	**<0.001***
**Glycine**	140.0 (41.4)	137.3 (38.3)	145.5 (46.5)	146.6 (49.9)	0.403	0.817
**Serine**	101.6 (35.7)	97.8 (32.3)	137.4 (58.5)	136.9 (63.5)	0.165	0.928
**Tyrosine**	27.2 (19.8)	28.0 (21.0)	16.7 (7.0)	18.7 (8.9)	0.658	0.014
**Non-coded AAs**						
**Citrulline**	10.6 (4.7)	10.9 (4.8)	20.9 (6.2)	20.5 (5.8)	0.410	0.424
**Ornithine**	14.4 (16.3)	15.6 (17.5)	13.6 (23.1)	18.6 (31.1)	0.370	0.071
**Taurine**	244.4 (94.3)	242.9 (91.3)	264.6 (103.2)	269.2 (111.3)	0.840	0.668
**Sum**						
**All FAAs**	2765.3 (668.7)	2707.3 (593.2)	3390.2 (681.8)	3350.0 (666.6)	0.253	0.543

FAA: free amino acid; EAA: essential amino acid; NEAA: non-essential amino acid. * *p*-value remains statistically significant following Bonferroni adjustment. Bonferroni-adjusted level of statistical significance is $\alpha = 0.05/22 = 0.002$. *P*-values < 0.002 are indicated in bold.

Table S5B. Total amino acid levels in human milk of mothers with or without an allergy.

TAA (μmol/L)	6 weeks Mean (SD)		6 months Mean (SD)		Analysis of differences at 6 weeks (*p*-value)	Analysis of differences at 6 months (*p*-value)
	Mothers without an allergy (n = 443)	Mothers with an allergy (n = 225)	Mothers without an allergy (n = 274)	Mothers with an allergy (n = 166)		
**EAAs**						
**Histidine**	1790.6 (262.2)	1805.2 (270.1)	1322.1 (197.3)	1334.9 (207.3)	0.646	0.221
**Isoleucine**	4013.1 (575.6)	4077.0 (646.8)	3380.2 (457.9)	3389.2 (464.2)	0.078	0.930
**Leucine**	8684.2 (1227.8)	8710.3 (1250.2)	6772.1 (902.8)	6833.8 (949.9)	0.759	0.127
**Lysine**	6367.2 (940.1)	6354.0 (969.2)	3948.8 (607.3)	3993.6 (653.1)	0.386	0.851
**Methionine**	1009.9 (207.3)	1011.5 (189.1)	749.7 (256.0)	752.5 (144.4)	0.763	0.292
**Phenylalanine**	2615.1 (439.4)	2605.0 (389.8)	2043.9 (282.3)	2082.9 (337.5)	0.506	0.132
**Threonine**	3700.5 (581.6)	3722.3 (576.0)	3345.0 (465.8)	3409.0 (567.5)	0.763	0.213
**Valine**	4654.0 (760.9)	4695.0 (747.8)	4164.4 (603.9)	4259.4 (663.0)	0.797	0.501
**NEAAs/Conditionally EAAs**						
**Alanine**	5349.3 (989.4)	5374.2 (990.5)	3938.8 (569.7)	4040.4 (733.9)	0.799	0.503
**Arginine**	2874.7 (1038.3)	3028.0 (1070.0)	2145.7 (545.6)	2140.4 (653.0)	0.805	0.567
**Asx**	8571.5 (1329.0)	8543.8 (1319.9)	6219.5 (858.6)	6283.7 (1039.3)	0.876	0.160
**Glx**	13469.5 (1622.0)	13502.7 (1655.9)	11517.4 (1448.8)	11426.8 (1702.5)	0.505	0.521
**Glycine**	3913.7 (720.3)	3888.6 (689.1)	2838.8 (451.1)	2921.1 (605.3)	0.662	0.130
**Serine**	5125.9 (828.5)	5115.5 (799.8)	4062.1 (571.6)	4152.9 (703.3)	0.918	0.884
**Tyrosine**	1895.6 (296.7)	1903.0 (298.9)	1498.3 (215.0)	1522.1 (238.3)	0.211	0.843
**Sum**						
**All TAAs**	74.0 (11.8)	74.3 (11.9)	57.9 (8.4)	58.5 (9.7)	0.867	0.473

TAA: total amino acid; EAA: essential amino acid; NEAA: non-essential amino acid; Glx: glutamate + glutamine; Asx: aspartate + asparagine. Bonferroni-adjusted level of statistical significance is $\alpha = 0.05/16 = 0.003$. *P*-values < 0.003 are indicated in bold.

Table S6A. Adjusted model results for associations between free amino acids in human milk and infant wheeze outcomes.

FAA	Wheeze					
	Transient wheeze		Persistent wheeze		Intermediate wheeze	
	6 weeks (n = 47) RR (95% CI)	6 months (n = 30) RR (95% CI)	6 weeks (n = 31) RR (95% CI)	6 months (n = 18) RR (95% CI)	6 weeks (n = 31) RR (95% CI)	6 months (n = 27) RR (95% CI)
**EAAs**						
**Histidine**	0.98 (0.95; 1.02)	1.00 (0.97; 1.03)	1.02 (0.98; 1.06)	1.02 (0.98; 1.07)	0.95 (0.89; 1.02)	**1.04 (1.01; 1.06)***
**Isoleucine**	0.98 (0.94; 1.02)	1.04 (0.97; 1.11)	1.00 (0.97; 1.04)	1.01 (0.91; 1.13)	0.97 (0.92; 1.02)	1.07 (0.99; 1.14)
**Leucine**	0.98 (0.95; 1.00)	1.02 (0.99; 1.05)	0.99 (0.96; 1.02)	1.02 (0.98; 1.06)	1.02 (1.00; 1.03)	1.04 (1.00; 1.09)
**Lysine**	0.99 (0.97; 1.00)	1.02 (0.99; 1.05)	0.98 (0.97; 1.00)	1.00 (0.96; 1.03)	1.00 (0.99; 1.03)	**1.04 (1.02; 1.06)***
**Methionine**	1.00 (0.98; 1.01)	1.00 (0.99; 1.02)	1.00 (0.99; 1.02)	1.01 (0.99; 1.02)	0.99 (0.97; 1.01)	1.02 (1.00; 1.03)
**Phenylalanine**	0.98 (0.93; 1.03)	1.09 (1.03; 1.15)‡	1.00 (0.97; 1.04)	1.05 (0.95; 1.16)	0.98 (0.93; 1.02)	1.04 (0.96; 1.12)
**Threonine**	1.00 (0.98; 1.01)	1.00 (0.99; 1.00)	1.00 (0.99; 1.01)	1.01 (1.00; 1.02)	1.00 (0.98; 1.01)	1.01 (1.01; 1.02)‡
**Tryptophan**	0.96 (0.92; 1.01)	1.01 (0.98; 1.04)	1.02 (0.98; 1.07)	0.99 (0.92; 1.06)	0.98 (0.91; 1.04)	0.99 (0.96; 1.03)
**Valine**	1.00 (0.99; 1.00)	1.01 (1.00; 1.03)	1.00 (0.99; 1.01)	1.00 (0.98; 1.02)	1.00 (0.99; 1.01)	1.01 (1.00; 1.02)
**NEAAs/conditionally EAAs**						
**Alanine**	1.00 (0.99; 1.01)	1.00 (1.00; 1.01)	1.00 (0.99; 1.01)	1.00 (0.97; 1.01)	1.00 (0.99; 1.01)	1.01 (1.00; 1.01)
**Arginine**	0.96 (0.92; 1.00)	1.06 (1.00; 1.11)	0.97 (0.93; 1.01)	0.93 (0.85; 1.00)	1.02 (0.98; 1.07)	1.04 (0.98; 1.10)
**Asparagine**	1.00 (0.98; 1.02)	1.00 (0.95; 1.05)	1.00 (0.98; 1.03)	0.96 (0.92; 1.00)	**0.95 (0.92; 0.98)***	1.04 (0.99; 1.11)

**Aspartate**	1.00 (0.98; 1.02)	0.99 (0.97; 1.00)	1.02 (1.00; 1.05)	1.00 (0.99; 1.02)	0.97 (0.95; 0.99)‡	1.00 (0.99; 1.01)
**Glutamate**	1.00 (1.00; 1.00)	1.00 (1.00; 1.00)	1.00 (1.00; 1.00)	1.00 (1.00; 1.00)	1.00 (1.00; 1.00)	1.00 (1.00; 1.00)
**Glutamine**	1.00 (1.00; 1.00)	1.00 (1.00; 1.00)	1.00 (1.00; 1.00)	1.00 (1.00; 1.00)	1.00 (0.99; 1.00)	1.00 (1.00; 1.00)
**Glycine**	1.00 (0.99; 1.01)	1.00 (0.99; 1.01)	1.01 (1.00; 1.02)	1.01 (1.00; 1.02)	0.99 (0.98; 1.00)	1.01 (1.00; 1.02)
**Serine**	0.99 (0.98; 1.01)	1.00 (0.99; 1.01)	1.00 (1.00; 1.01)	1.01 (1.00; 1.02)	0.98 (0.96; 1.00)‡	1.01 (1.00; 1.01)‡
**Tyrosine**	1.00 (0.98; 1.02)	1.01 (0.97; 1.06)	1.01 (0.99; 1.03)	1.02 (0.93; 1.10)	0.96 (0.94; 0.99)‡	1.06 (1.00; 1.12)
			**Non-coded AAs**			
**Citrulline**	0.96 (0.91; 1.02)	1.05 (0.97; 1.12)	1.01 (0.94; 1.09)	0.99 (0.92; 1.08)	0.88 (0.78; 0.99)	1.04 (0.95; 1.14)
**Ornithine**	0.99 (0.96; 1.02)	0.99 (0.97; 1.01)	1.02 (1.00; 1.04)	1.02 (1.00; 1.03)	0.98 (0.94; 1.02)	1.01 (1.01; 1.02)
**Taurine**	1.00 (1.00; 1.00)	1.00 (1.00; 1.00)	1.00 (0.99; 1.00)	1.00 (0.99; 1.01)	1.00 (1.00; 1.01)	1.00 (0.99; 1.00)
			**Sum**			
**All FAAs**	1.00 (1.00; 1.00)	1.00 (1.00; 1.00)	1.00 (1.00; 1.00)	1.00 (1.00; 1.00)	1.00 (1.00; 1.00)	1.00 (1.00; 1.00)

FAA: free amino acid; EAA: essential amino acid; NEAA: non-essential amino acid; RR: risk ratio. ‡ $p < 0.01$, * p-value remains statistically significant following Bonferroni adjustment. Bonferroni adjusted level of statistical significance is $\alpha = 0.05/22 = 0.0023$. P-values < 0.0023 are indicated in bold. Models were adjusted for maternal pre-pregnancy body mass index (BMI), maternal age, infant sex, maternal education and smoking status.

Table S6B. Adjusted model results for associations of free amino acids in human milk and infant atopic disease and lower respiratory tract infections.

FAA	Atopic disease (AD)						Lower respiratory tract infections	
	Parent-reported AD		Paediatrician-reported AD		Both parent- and paediatrician-reported AD			
	6 weeks (n = 44) RR (95% CI)	6 months (n = 27) RR (95% CI)	6 weeks (n = 64) RR (95% CI)	6 months (n = 37) RR (95% CI)	6 weeks (n = 73) RR (95% CI)	6 months (n = 44) RR (95% CI)	6 weeks (n = 151) RR (95% CI)	6 months (n = 108) RR (95% CI)
**EAAs**								
**Histidine**	0.98 (0.95; 1.02)	0.98 (0.92; 1.04)	0.98 (0.95; 1.01)	1.01 (0.98; 1.04)	0.97 (0.93; 1.02)	0.98 (0.92; 1.05)	0.99 (0.96; 1.01)	1.01 (1.00; 1.03)
**Isoleucine**	1.01 (0.98; 1.05)	0.91 (0.78; 1.06)	1.01 (0.98; 1.04)	0.99 (0.91; 1.07)	1.02 (0.98; 1.06)	0.89 (0.72; 1.10)	0.97 (0.95; 0.99)	1.03 (0.99; 1.06)
**Leucine**	1.01 (0.98; 1.03)	0.93 (0.87; 1.00)	1.00 (0.98; 1.02)	0.97 (0.92; 1.02)	1.00 (0.97; 1.04)	0.92 (0.84; 1.01)	1.00 (0.98; 1.01)	1.01 (0.99; 1.03)
**Lysine**	1.01 (1.00; 1.02)	0.99 (0.96; 1.02)	1.01 (0.99; 1.02)	1.00 (0.98; 1.03)	1.01 (1.00; 1.03)	0.97 (0.95; 1.00)	1.00 (0.99; 1.01)	1.01 (1.00; 1.02)
**Methionine**	1.00 (0.98; 1.02)	0.98 (0.96; 1.00)	1.00 (0.98; 1.01)	0.99 (0.98; 1.01)	1.01 (0.99; 1.02)	0.98 (0.95; 1.00)	0.99 (0.98; 1.00)	1.00 (0.99; 1.01)
**Phenylalanine**	1.00 (0.97; 1.04)	0.95 (0.81; 1.11)	0.99 (0.96; 1.02)	0.96 (0.87; 1.07)	1.00 (0.96; 1.04)	0.95 (0.78; 1.15)	1.00 (0.97; 1.04)	0.98 (0.96; 1.00)
**Threonine**	1.01 (0.99; 1.02)	0.98 (0.96; 1.01)	1.00 (0.99; 1.02)	1.00 (0.99; 1.01)	1.00 (0.99; 1.02)	0.98 (0.96; 1.01)	0.99 (0.98; 1.00)	1.00 (1.00; 1.01)
**Tryptophan**	1.04 (1.00; 1.08)	1.00 (0.96; 1.04)	1.02 (0.98; 1.06)	1.00 (0.97; 1.04)	1.04 (0.99; 1.08)	1.00 (0.96; 1.05)	0.99 (0.96; 1.01)	0.99 (0.97; 1.02)
**Valine**	0.99 (0.99; 1.00)	0.97 (0.95; 1.00)	1.00 (0.99; 1.00)	0.99 (0.98; 1.01)	0.99 (0.98; 1.00)	0.97 (0.94; 1.01)	1.00 (1.00; 1.00)	1.01 (1.00; 1.02)
**NEAAs/conditionally EAAs**								
**Alanine**	1.00 (0.99; 1.00)	1.00 (0.99; 1.00)	1.00 (0.99; 1.00)	1.00 (1.00; 1.01)	1.00 (0.99; 1.00)	1.00 (0.99; 1.00)	1.00 (0.99; 1.00)	1.00 (1.00; 1.01)
**Arginine**	1.04 (1.00; 1.08)	0.95 (0.89; 1.01)	1.01 (0.97; 1.04)	1.00 (0.95; 1.06)	1.03 (0.98; 1.08)	0.93 (0.85; 1.02)	1.00 (0.98; 1.02)	1.03 (1.00; 1.05)
**Asparagine**	1.00 (0.97; 1.02)	0.94 (0.87; 1.01)	0.99 (0.96; 1.01)	0.96 (0.91; 1.02)	0.99 (0.96; 1.02)	0.93 (0.85; 1.01)	0.98 (0.97; 1.00)	1.02 (0.99; 1.04)
**Aspartate**	1.01 (0.98; 1.03)	1.00 (0.99; 1.01)	1.00 (0.98; 1.01)	1.00 (0.99; 1.01)	1.01 (0.99; 1.04)	0.99 (0.98; 1.01)	0.99 (0.98; 1.00)	1.00 (1.00; 1.01)
**Glutamate**	1.00 (1.00; 1.00)	0.99 (0.99; 0.99)‡	1.00 (1.00; 1.00)	0.99 (0.99; 0.99)	1.00 (1.00; 1.00)	0.99 (0.99; 0.99)‡	1.00 (1.00; 1.00)	1.00 (1.00; 1.00)

**Glutamine**	1.00 (1.00; 1.00)	1.00 (1.00; 1.00)	1.00 (1.00; 1.00)	1.00 (1.00; 1.00)	1.00 (1.00; 1.00)	1.00 (1.00; 1.00)	1.00 (1.00; 1.00)	1.00 (1.00; 1.00)
**Glycine**	1.00 (0.99; 1.01)	0.99 (0.97; 1.01)	0.99 (0.99; 1.00)	1.00 (0.99; 1.01)	1.00 (0.99; 1.00)	0.99 (0.97; 1.01)	0.99 (0.99; 1.00)	1.00 (1.00; 1.01)
**Serine**	1.01 (1.00; 1.02)	1.00 (0.99; 1.01)	1.00 (0.99; 1.01)	1.00 (0.99; 1.01)	1.01 (0.99; 1.02)	1.00 (0.99; 1.01)	1.00 (0.99; 1.00)	1.00 (1.00; 1.01)
**Tyrosine**	1.01 (0.99; 1.03)	0.91 (0.81; 1.02)	1.00 (0.98; 1.02)	0.96 (0.90; 1.03)	1.01 (0.99; 1.03)	0.90 (0.79; 1.03)	0.99 (0.98; 1.00)	1.01 (0.98; 1.03)
**Non-coded AAs**								
**Citrulline**	1.03 (0.97; 1.10)	0.96 (0.88; 1.05)	0.99 (0.92; 1.07)	0.97 (0.90; 1.04)	1.05 (0.96; 1.14)	0.99 (0.89; 1.09)	0.97 (0.93; 1.02)	1.00 (0.96; 1.04)
**Ornithine**	0.99 (0.95; 1.04)	1.00 (0.99; 1.02)	0.98 (0.95; 1.02)	1.01 (1.00; 1.02)	1.00 (0.96; 1.05)	1.00 (0.99; 1.02)	1.00 (0.98; 1.02)	1.01 (1.00; 1.01)
**Taurine**	1.00 (1.00; 1.00)	1.00 (0.99; 1.01)	1.00 (1.00; 1.01)	1.00 (1.00; 1.01)	1.00 (1.00; 1.01)	1.00 (0.99; 1.01)	1.00 (1.00; 1.00)	1.00 (1.00; 1.00)
**Sum**								
**All FAAs**	1.00 (1.00; 1.00)	0.99 (0.99; 0.99)‡	1.00 (1.00; 1.00)	1.00 (1.00; 1.00)	1.00 (1.00; 1.00)	0.99 (0.99; 0.99)‡	1.00 (1.00; 1.00)	1.00 (1.00; 1.00)

FAA: free amino acid; EAA: essential amino acid; NEAA: non-essential amino acid; RR: risk ratio. ‡ $p < 0.01$, * p-value remains statistically significant following Bonferroni adjustment. Bonferroni-adjusted level of statistical significance is $\alpha = 0.05/22 = 0.002$. P-values < 0.002 are indicated in bold. Models were adjusted for maternal pre-pregnancy body mass index (BMI), maternal age, infant sex, maternal education and smoking status.

Table S6C. Adjusted model results for associations between free amino acids in human milk with infant food allergy.

FAA	Food allergy	
	**6 weeks (n = 13)** **RR (95% CI)**	**6 months (n = 7)** **RR (95% CI)**
**EAAs**		
**Histidine**	1.02 (0.80; 1.29)	1.00 (0.99; 1.01)
**Isoleucine**	0.99 (0.82; 1.17)	1.00 (0.97; 1.03)
**Leucine**	0.99 (0.86; 1.14)	1.00 (0.99; 1.01)
**Lysine**	0.98 (0.83; 1.15)	1.00 (0.99; 1.01)
**Methionine**	0.93 (0.85; 1.02)	1.00 (0.99; 1.01)
**Phenylalanine**	1.01 (0.86; 1.18)	1.00 (0.97; 1.03)
**Threonine**	0.95 (0.89; 1.03)	1.00 (1.00; 1.00)
**Tryptophan**	0.97 (0.78; 1.22)	1.00 (0.99; 1.01)
**Valine**	1.00 (0.97; 1.03)	1.00 (0.99; 1.01)
**NEAAs/conditionally EAAs**		
**Alanine**	0.99 (0.96; 1.02)	1.00 (1.00; 1.00)
**Arginine**	0.92 (0.86; 0.99)†	1.00 (0.98; 1.02)
**Asparagine**	**0.70 (0.58; 0.84)***	1.00 (0.98; 1.02)
**Aspartate**	0.97 (0.92; 1.03)	1.00 (1.00; 1.00)
**Glutamate**	0.94 (0.83; 1.04)	1.00 (1.00; 1.00)
**Glutamine**	0.85 (0.69; 1.02)	1.00 (1.00; 1.00)
**Glycine**	0.98 (0.94; 1.02)	1.00 (1.00; 1.00)
**Serine**	0.97 (0.93; 1.01)	1.00 (1.00; 1.00)
**Tyrosine**	0.93 (0.80; 1.07)	1.00 (0.98; 1.02)

**Non-coded AAs**		
**Citrulline**	0.85 (0.71; 0.98)†	1.00 (0.98; 1.03)
**Ornithine**	0.98 (0.82; 1.18)	1.00 (0.99; 1.01)
**Taurine**	0.99 (0.97; 1.02)	1.00 (1.00; 1.00)
**Sum**		
**All FAAs**	0.94 (0.84; 1.05)	1.00 (1.00; 1.00)

FAA: free amino acid; EAA: essential amino acid; NEAA: non-essential amino acid; RR: risk ratio. † $p < 0.05$, * p-value remains statistically significant following Bonferroni adjustment. Bonferroni-adjusted level of statistical significance is $\alpha = 0.05/23 = 0.002$. P-values < 0.002 are indicated in bold. Models were adjusted for maternal pre-pregnancy body mass index (BMI), maternal age, infant sex, maternal education and smoking status.
